# Visualization of Multi-indication Randomized Control Trial Evidence to Support Decision Making in Oncology: A Case Study on Bevacizumab

**DOI:** 10.1177/0272989X261430333

**Published:** 2026-03-31

**Authors:** Sumayya Anwer, Janharpreet Singh, Sylwia Bujkiewicz, Anne Thomas, Richard Adams, Elizabeth Smyth, Pedro Saramago, Stephen Palmer, Marta O. Soares, Sofia Dias

**Affiliations:** Centre for Reviews and Dissemination, University of York, York, UK; Biostatistics Research Group, Department of Population Health Sciences, University of Leicester, Leicester, UK; Biostatistics Research Group, Department of Population Health Sciences, University of Leicester, Leicester, UK; Leicester Cancer Research Centre, University of Leicester, Leicester, UK; Cardiff University, Cardiff, UK; Velindre Cancer Centre, Cardiff, UK; Oxford NIHR Biomedical Research Centre, Churchill Hospital, Oxford, UK; Centre for Health Economics, University of York, York, UK; Health Economics and Market Access, Evidera by PPD, Thermo Fisher Scientific, UK; Centre for Health Economics, University of York, York, UK; Centre for Health Economics, University of York, York, UK; Centre for Reviews and Dissemination, University of York, York, UK

**Keywords:** meta-analysis, health technology assessment, oncology, multi-indication drugs

## Abstract

**Background:**

As an increasing number of oncology drugs are licensed for multiple indications, sharing information across indications may help improve the precision of estimates for a target indication where evidence may be immature. Visualizing the accumulation of evidence and its characteristics across all indications can help inform policy makers as to whether multi-indication synthesis methods should be considered and guide expert elicitation on appropriate cross-indication assumptions.

**Methods:**

The multi-indication oncology drug bevacizumab was selected as a case study. We used visualization methods including timeline, ridgeline, and split-violin plots to display evidence and synthesis results across 7 licensed cancer types, focusing on the evidence on overall and progression-free survival and the display of results from models with and without information sharing.

**Results:**

The proposed displays allow for visualization of key characteristics of the evidence to support the assessment of heterogeneity within and across indications and inform the feasibility of information-sharing models.

**Limitations:**

The lack of consistent reporting of data in trial reports limits the visualization of some study characteristics. Tradeoffs between plot readability and the level of detail to include were required.

**Conclusions:**

Clear graphical representations of the evolution and accumulation of evidence and synthesis results can provide a better understanding of the entire multi-indication evidence base, which can inform judgments regarding the appropriate use of data within and across indications. Interactive plots could help overcome some of the current limitations.

**Implications:**

The proposed displays should be used to facilitate discussion with experts on the judgments required to assess the feasibility of using information-sharing methods to improve the estimation of relative treatment effects in evidence synthesis approaches and health technology assessment.

**Highlights:**

- An increasing number of oncology drugs are licensed for multiple indications; we developed visualization methods for multi-indication evidence that consider key characteristics unique to oncology.
- Graphical displays can be used to show the evolution of evidence within and across multiple indications.
- Clear evidence visualizations can be used as a tool to support evidence synthesis approaches, support policy makers, or guide expert elicitation.

## Introduction

An increasing number of oncology drugs are licensed for multiple indications, typically sequentially. Thus, a drug is initially licensed for one indication, and over time, its license is extended to include additional indications. However, health technology assessment (HTA) bodies generally appraise drugs for 1 indication at a time (the “target” indication) and ignore evidence from other indications. The use of often immature evidence (with few events observed due to short follow-up time) from only 1, or very few, indication-specific trials can result in uncertain treatment effect estimates. Sharing evidence from previously licensed indications in similar disease areas can strengthen estimates for the target indication and lead to better decisions.

Bayesian information-sharing models have been proposed to pool treatment effects across, as well as within, indications, allowing for both between and within indication variation, and increase precision of estimates in the target indication.^1–4^ However, analyses need to be supported by judgments about the feasibility and appropriateness of combining evidence across indications as, in the presence of within and between indication heterogeneity, precision increases can be limited and bias may result if an outlier indication (that is, an indication that is very different from the other included indications) is included; however, outlying indications cannot always be identified statistically.^5^ Thus, expert judgment is required as to which indications (if any) should be pooled and whether heterogeneity or outlier indications are likely to be present.

To support the required judgments on the feasibility of information-sharing methods and evaluations of heterogeneity, effective visualization techniques are needed to understand the multi-indication evidence base within and across indications. The plausibility of evidence synthesis results and the effect of information sharing also need to be assessed before results can be used in decision making.

Evidence maps are visual tools that can be used to summarize existing evidence by displaying key characteristics such as the number and type of studies, study design, populations, interventions, comparators, or outcomes to guide stakeholders to high-quality research, inform research priority setting, and help define the focus of evidence synthesis^6^ or to identify and highlight evidence gaps.^7^ Data visualizations may be static or interactive.^8^ Within a health care context, evidence maps have been used to support decision making,^9^ identify gaps in evidence,^10^ and understand the extent and distribution of evidence.^8,11,12^

However, attributes of multi-indication oncology evidence introduce challenges for visually displaying evidence and synthesis results that have not been addressed in current literature. Typically, 2 related time to event outcomes, progression-free survival (PFS) and overall survival (OS), are of interest, with studies reporting 1 or both outcomes at multiple (interim and final), potentially different, time points. In addition, relevant comparators may differ across indications, and different levels of heterogeneity may be expected depending on which studies and which indications are combined.

We propose displays of multi-indication evidence and synthesis results to support feasibility assessment and evaluation of potential heterogeneity when considering the use of information-sharing models using bevacizumab (first licensed as Avastin^®^) as a case study. In the next section, we describe the case study. The methods section motivates the key features of oncology evidence to display, describes the evidence synthesis methods under consideration, and describes the visualization tools proposed. The results section implements the proposed evidence in the case study. We conclude with a discussion and suggestions for further developments and extensions.

## Bevacizumab Case Study

Bevacizumab was the first available angiogenesis inhibitor therapy and was selected as a case study due to its extensive evidence base across multiple cancer indications accumulated over a period of more than 20 years. It was initially licensed for the treatment of metastatic colorectal cancer in combination with chemotherapy in the United States in 2004 and the European Union in 2005.^13^ The National Institute for Health and Care Excellence (NICE) in the United Kingdom undertook the first UK HTA appraisal of bevacizumab for the treatment of metastatic colorectal cancer in 2007.

To determine the indications for which bevacizumab is approved, we used the summary of product characteristic (SmPC) for Avastin^®^, issued by the European Medicines Agency,^14^ which identified 7 cancer types: breast cancer, cervical cancer, colorectal cancer, glioblastoma, non-small-cell lung cancer, renal cell carcinoma, and ovarian cancer (used here to refer to ovarian, fallopian tube, and primary peritoneal cancers, collectively).

Searching for evidence on these indications was conducted in 2 stages: first, we searched for all relevant comparative phase II or phase III randomized controlled trials (RCTs) of bevacizumab included in NICE appraisals, the SmPC for Avastin^®^, or Cochrane reviews on the 7 licensed cancer indications. This was followed by a second search on the clinicaltrials.gov database^15^ for phase III Avastin^®^ trials that were either complete or had been terminated prior to completion. Any trials not previously identified were considered. We also included studies from 2 relevant systematic reviews^13,16^ already known to us. The identification of studies is depicted in Supplementary Figure S1, with study details provided in Supplementary Table S1.

We included only oncology studies in the metastatic/advanced setting in which the treatment effect for bevacizumab could be isolated from any background chemotherapies or other targeted therapies administered during the trial. We excluded studies in nonlicensed indications and noncancer therapeutic areas (e.g., macular degeneration). Studies in which bevacizumab was administered in an adjuvant or neoadjuvant setting were excluded as the treatment effect of bevacizumab was expected to differ substantially from the advanced/metastatic setting.

Data extraction focused on OS and PFS, the time-to-event outcomes typically of primary interest for oncology HTA and used in supporting economic models. For each selected trial, we retrieved all available publications and extracted data on the OS and PFS relative treatment effects (RTEs) as hazard ratios (HRs) with 95% confidence intervals (CIs) comparing bevacizumab against alternative treatments for all interim and final data points. Details of the data extraction process are included in Supplementary Section A-II. The final dataset consisted of 41 unique trials across 7 cancer types (Supplementary Tables S2 and S3).

## Methods

To support the assessment of the feasibility of fitting information-sharing models to strengthen inferences for a given target indication, we propose plots to visualize the quantity and maturity of the evidence available across indications (see the following sub-section). To support judgments on homogeneity and consistency of RTEs within and across indications, which in turn inform judgments on whether any precision gains are expected from using information-sharing models, plots displaying the RTEs across indications are proposed (see the sub-section on visualizing outcome data). Plots to facilitate the comparison of results from standard (nonsharing) and information-sharing synthesis models are considered in the sub-section on evidence synthesis.

First, we consider displays to represent all the evidence available. Then we consider breast cancer as the target indication and propose displays to assess the feasibility and potential impact of multi-indication synthesis at 2 early time points, 2009, 2012, and the latest time point 2021, when all evidence on bevacizumab for all indications is available. The first 2 time points were selected to show the effect on estimates of RTE from increasing within and across indication data availability. At time point 1 (2009), the evidence for breast cancer consisted of only 5 studies reporting on OS, but evidence from 10 studies from 3 other indications was also available; for PFS, there was evidence from 6 breast cancer studies and 12 studies from 3 other indications. Available evidence for OS at time point 2 (2012) included the same 5 breast cancer studies available at time point 1, but 18 studies from 6 other indications had reported data for OS, with 7 breast cancer studies reported on PFS along with 22 studies from 6 other indications. We assume studies from the other indications available are appropriate for inclusion in an information-sharing model. Ovarian cancer is considered as a second target indication, for illustration, with time points defined as time point 1 (2011) when evidence for OS (PFS) for ovarian cancer consisted only of 3 (4) studies, but 17 (21) studies from 4 other indications are also available; time point 2 (2015) when evidence for OS (PFS) includes 4 (5) ovarian cancer studies and a further 27 (33) studies from 6 indications.

### Visualizing the Evolution of Evidence

Timeline plots show how trial evidence accumulates over time and the impact of accumulating evidence on estimated treatment effects.^13,17,18^ We propose a timeline (or time trend^19^) plot, with time represented on the horizontal axis. The start of each trial is depicted by a small vertical line with a superimposed square, weighted according to the trial’s overall sample size (the size of the square increases with the number of patients in the trial). Larger, more precise studies are represented by larger squares, following the convention used for forest plots presenting the results of meta-analyses. As per current practice in the field of meta-analysis, larger, more precise studies are here represented by larger squares, to bring focus to studies that may be more influential for meta-analysis and any variations across indications.^20,21^ If instead the aim was to emphasize uncertainty, larger squares could be used to represent smaller studies.

Time points at which HRs for different outcomes are reported are denoted by different symbols. A horizontal line, depicting the duration of the trial, is used to join all time points. Key trial characteristics, such as type of comparator, setting, design, or risk of bias, can be noted by adding labels or different colors or line styles for greater visual impact. When outcome reporting dates are too close together, markers on the timeline plots overlap, making them hard to distinguish. A small gap between 2 reporting points is added to improve visibility in the plots.

Displaying the accumulation of evidence across all indications over time can inform judgments on whether sufficient evidence is available to justify multi-indication synthesis for the target indication at a given time point.^4^ Highlighting key trial characteristics can also provide evidence on potential heterogeneity within and between indications.

#### Maturity of evidence

The maturity of time-to-event data relates to how complete the trial is in terms of observing the event(s) of interest in all trial participants at the time of reporting, which is affected by follow-up time and censoring. Meaningful increases in precision for the target indication are expected only when sufficiently mature evidence from other indications is available. To assess the feasibility of information-sharing methods, it is useful to consider the maturity of evidence across all indications.

However, there is no single accepted metric of maturity. Often a quantification of length of follow-up (typically the median) in each study arm or across arms is reported, but in many trials, it is unclear how this was defined.^22^ In general, evidence can be considered more mature if it is reported at a later time point.^23^ However, when comparing the maturity of evidence across indications, a longer follow-up time in one indication may not necessarily translate to more observed survival events if prognosis is more favorable than in other indications. For a given follow-up duration, an index of completeness using information from digitized Kaplan–Meier (KM) curves has been proposed.^24^ Although this provides a measure of data completeness that is comparable across indications, it is not usually reported in trials, and the data collection burden of digitizing all KM curves for its calculation is considerable.

Here we define maturity as the proportion of patients who experience an event, relative to the total number of patients included in the trial, which can be compared across indications.^25^ These proportions are represented by weighted circles at the point at which each outcome was reported for each trial, with larger circles indicating more mature evidence. When choosing how to weight the circles, it is important to consider there is a limit to how circles of different diameters appear distinct to the naked eye, so scaling may need to be adjusted to ensure adequate visualization. Focus is on the relative sizes of the circles, within and across indications, to assess differences in maturity across the whole evidence base, rather than on the absolute size of each circle. Precise values can be given in tables.

#### Uncertainty/precision

The uncertainty associated with the estimates from each trial can influence how much increase in precision of the RTEs for the target indication is expected from multi-indication synthesis and help inform feasibility. Uncertainty can be represented by the width of the 95% CI (or credible interval, CrI), calculated as the difference between the upper and lower limits, where a smaller width indicates more precision in the estimate and less uncertainty in the RTE, or it can be expressed relative to the magnitude of the RTE on the log scale as $\frac{\mathrm{SE}}{\left|\ln(\mathrm{HR})\right|}$ , analogous to the coefficient of variation,^26^ which can be meaningfully compared across indications, where SE is the standard error of the ln(HR). The smaller the ratio, the more precise (less uncertain) the estimate. Here we represent precision in a modified timeline plot by circles at the point at which the outcome was reported for each trial weighted by the inverse of this ratio. An increase in precision (less uncertainty) is depicted by an increase in the area of the circles. It is important to note when scaling as a function of area that the size of the circles is not precisely aligned with differences in precision, as the area of a circle does not increase linearly. Alternatively, the size of the circles can be varied by weighting the radius of the circle by the uncertainty, depending on the aims of the display and the parameters being visualized. The relative sizes of the circles across studies can be used as a guide, to judge whether using evidence from other indications has the potential to strengthen estimates in the target indication; for example, if the precision of studies in other indications is lower than for studies in the target indication, there may not be value in adding them to the synthesis. In addition, the presence of extreme values can lead to difficulties in perceiving differences in circle sizes for other values. In such cases, it may be necessary to fix a maximum (or minimum) circle diameter and assign all points with higher (or lower) precision the same fixed size to retain comparability of the other circles with the exact values presented in tables.

### Visualizing Outcome Data

Traditionally, results of trials and pooled estimates generated from meta-analyses have been presented as forest plots,^19,21^ with point estimates represented as circles or squares and their corresponding 95% CIs or CrIs as a line between the lower and upper bounds. However, forest plots give the false perception that all points within the interval are supported equally by the evidence and that values outside the interval are not possible.^27^ Ridgeline plots^28^ efficiently display densities for multiple groups as partially overlapping plots sharing a common scale on the horizontal axis providing an overview of the complete outcome distribution. They are particularly useful to display multiple groups with a clear pattern (e.g., rankings or ordering) to represent, and using separate plots would take up too much space.

To highlight differences between outcomes within and across indications at different time points and inform judgments about potential heterogeneity or outliers within and across indications, we propose ridgeline plots to display the density of the final reported RTE (assuming a normal distribution for the reported ln[HR] with variance calculated from the 95% CIs) for both PFS and OS for each indication. The timeline is now in the vertical axis, instead of the horizontal axis, to align with familiar presentations of evidence in a meta-analysis context (where typically the vertical axis is labeled with the study name) and with how densities are typically represented (with the relevant scale on the *x*-axis).

### Evidence Synthesis

In HTA, for a single target indication with 2 or more studies available, a meta-analysis is usually conducted to pool the results of relevant studies within that indication and to estimate the overall RTE.^29,30^ Common (also known as fixed) or random effects models can be used when RTEs estimated by the different studies are expected to be equal or heterogeneous, respectively. The standard meta-analysis model does not share information across indications and can be considered the reference model in HTA. In the multi-indication context, we will term this the independent parameter (IP) meta-analysis model, where the treatment effect for an indication is formed by pooling only within indication RTEs, with no information sharing across indications.

Where sharing of information across indications has been deemed useful and feasible, information-sharing models allowing for different levels of sharing across indications can be used.^1–4^ The selection of appropriate models to fit should be done on a case-by-case basis, following inspection of the available evidence, supported by expert opinion of what are realistic assumptions and which indications should be included in the sharing model. Regardless of the sharing model(s) selected, results should be compared with the IP model to assess their plausibility and understand any precision gains or shifts in point estimates (which might indicate bias), before results are used to inform decisions.

For illustration, we consider here the common parameter (CP) multi-indication meta-analysis model, which assumes a common treatment effect across indications so a single effect is estimated for all indications (complete borrowing), while allowing for within indication heterogeneity (i.e., random effects meta-analysis model within each indication).^31,32^ For detailed specification of the IP and CP models, see Supplementary Section B-I.

A cumulative meta-analysis framework,^33,34^ in which a new meta-analysis is conducted when a study reports its final outcome, is used to explore how RTEs change with the accumulation of evidence over time. Results reflect all evidence available up to that point in time and change as additional studies report results. For the IP model, the cumulative meta-analyses include only within indication information, whereas for the CP model, evidence that has accumulated on other indications up to that time point is also included through the sharing assumptions.

Ridgeline plots are used to display the results of cumulative IP meta-analyses performed for each indication, with the vertical axis representing time. At each time point at which a trial has reported its final result the density for the ln(HR) reported in the study and the density of the final RTE from the cumulative meta-analysis at that time point are plotted using different shading and labeled with the name of the new trial being included.

Split-violin plots are proposed to compare the posterior distributions from the IP and CP models for each of the outcomes. Violin plots^35^ were proposed as a modification to the box plot to show the underlying density of the data, mirrored across a central line where the observed data points or summary statistics can be displayed. Split-violin plots represent 2 different densities on each side of the central line to compare 2 distributions.^36^

For a given target indication where multi-indication synthesis is being considered at 3 different time points, ridgeline and split-violin plots are proposed to illustrate results at each time point from models with and without information sharing.

### Implementation

All plots were created in R version 4.4.1^37^ using the ggplot2^38^ v3.5.1, ggridges,^28^ v0.5.6 and introdataviz^36^ v0.0.0.9003 packages. Meta-analyses were conducted in a Bayesian framework using Markov chain Monte Carlo (MCMC) simulation, implemented using OpenBUGS^39^ code adapted from Singh et al.^4^ through the R2OpenBUGS^40^ package. Posterior density plots for the results of all meta-analyses were created by directly plotting the output from the MCMC simulations.

## Results: Evidence Mapping in the Bevacizumab Case Study

### Displaying the Evolution of Evidence

**Figure 1:**
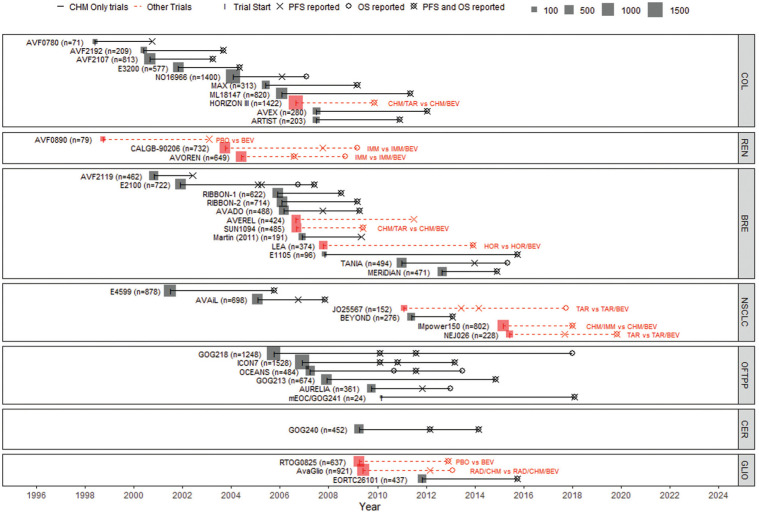
Timeline plot of all licensed indications for bevacizumab. BEV, bevacizumab; BRE, breast cancer; CER, cervical cancer; CHM, chemotherapy; COL, colorectal cancer; GLIO, glioblastoma; HOR, hormonal therapy; IMM, immunotherapy; NSCLC, non-small-cell lung cancer; OFTPP, ovarian, fallopian tube and primary peritoneal cancer; OS, overall survival; PBO, placebo; PFS, progression-free survival; RAD, radiotherapy; REN, renal cell carcinoma; TAR, targeted therapy. Squares at the start point are weighted according to the trial size.

Figure 1 shows the timeline plot summarizing the bevacizumab evidence (see the previous section methods). Indications are presented in chronological order, starting with the indication with the earliest trial start date at the top. Time points at which the HRs for OS and PFS were reported are denoted by a circle and a cross, respectively. Trials did not always report OS and PFS at the same time points, with PFS results typically being reported earlier. Most studies compared bevacizumab in combination with chemotherapy to chemotherapy alone. Following clinical advice, we decided not to differentiate between the different chemotherapy regimens, as the additive effect of bevacizumab (that is bevacizumab in addition to another therapy compared to that therapy alone) would be reasonably independent of the base therapy, but comparisons against non-chemotherapy treatments are shown as dashed red lines along with annotations specifying the comparator. Later trials compare the effectiveness of bevacizumab to targeted therapies more often than chemotherapy. However, clinical advice was that RTEs were not expected to meaningfully differ across different comparators, so all studies could be included in a multi-indication synthesis.

**Figure 2:**
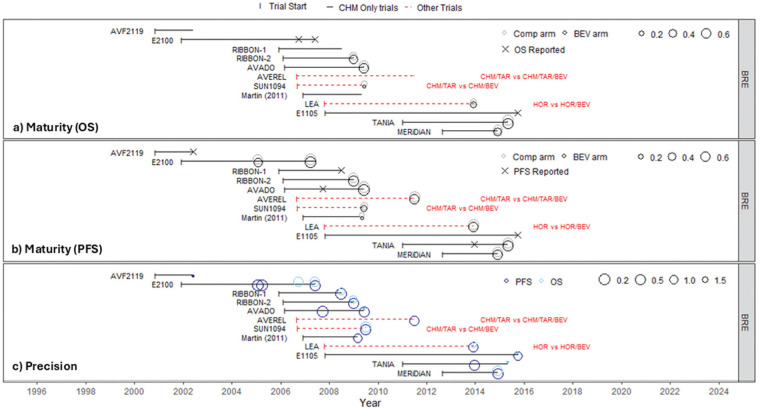
Modified timeline plots for breast cancer showing (a) maturity of OS evidence, (b) maturity of PFS evidence, and (c) precision of OS and PFS evidence. Precisions under 1/2 = 0.5 are plotted as solid points instead of sized circles. BEV, bevacizumab; CHM, chemotherapy; CI, confidence interval; Comp, comparator; HOR, hormonal therapy; IMM, immunotherapy; NSCLC, non-small-cell lung cancer; OS, overall survival; PBO, placebo; PFS, progression-free survival; TAR, targeted therapy. An arbitrary gap of 3 to 4 months was added to improve visibility, where necessary. In Figure 2a–c, a gap of 4 months was introduced between AVADO and RIBBON-2 by moving the marker for the final AVADO data point 2 months later and 2 months earlier for RIBBON-2. In 2(c), a gap of 3 months was introduced between SUN1094 and Martin (2011) by moving the marker for SUN1094 1 months later and Martin (2011) 2 months later.

Figure 2 presents modified timeline plots, displaying data maturity and precision using breast cancer as an example. Equivalent plots showing all indications are included in Supplementary Section D (Figures S2–S4) with all plotted values presented in Supplementary Section C. In Figure 2a and b, circles for both treatment arms are weighted according to the maturity of OS and PFS evidence, respectively, at each reporting time point. Crosses indicate points at which the outcomes were reported but maturity could not be calculated. Comparing evidence maturity across indications (Supplementary Figures S2 and S3) can provide insights into the potential benefits of using an information-sharing model; however, in this case, interpretation is hindered by limited reporting. Where maturity can be calculated, OS evidence is more mature for the comparator arm than for the bevacizumab arm, but PFS maturity is similar across arms within a trial. Across studies, levels of maturity of PFS are comparable, whereas maturity for OS differs.

Figure 2c shows that PFS estimates at a given time point are generally more precise than OS estimates, as more PFS events are expected to have occurred. Supplementary Figure S4 shows that, at earlier time points when only a few breast cancer studies have reported OS (2009, 2012), other indications already have OS data reported with substantial precision, suggesting that sharing information across indications may improve estimation of the ln(HR) of OS for breast cancer.

### Displaying Outcome Data

**Figure 3:**
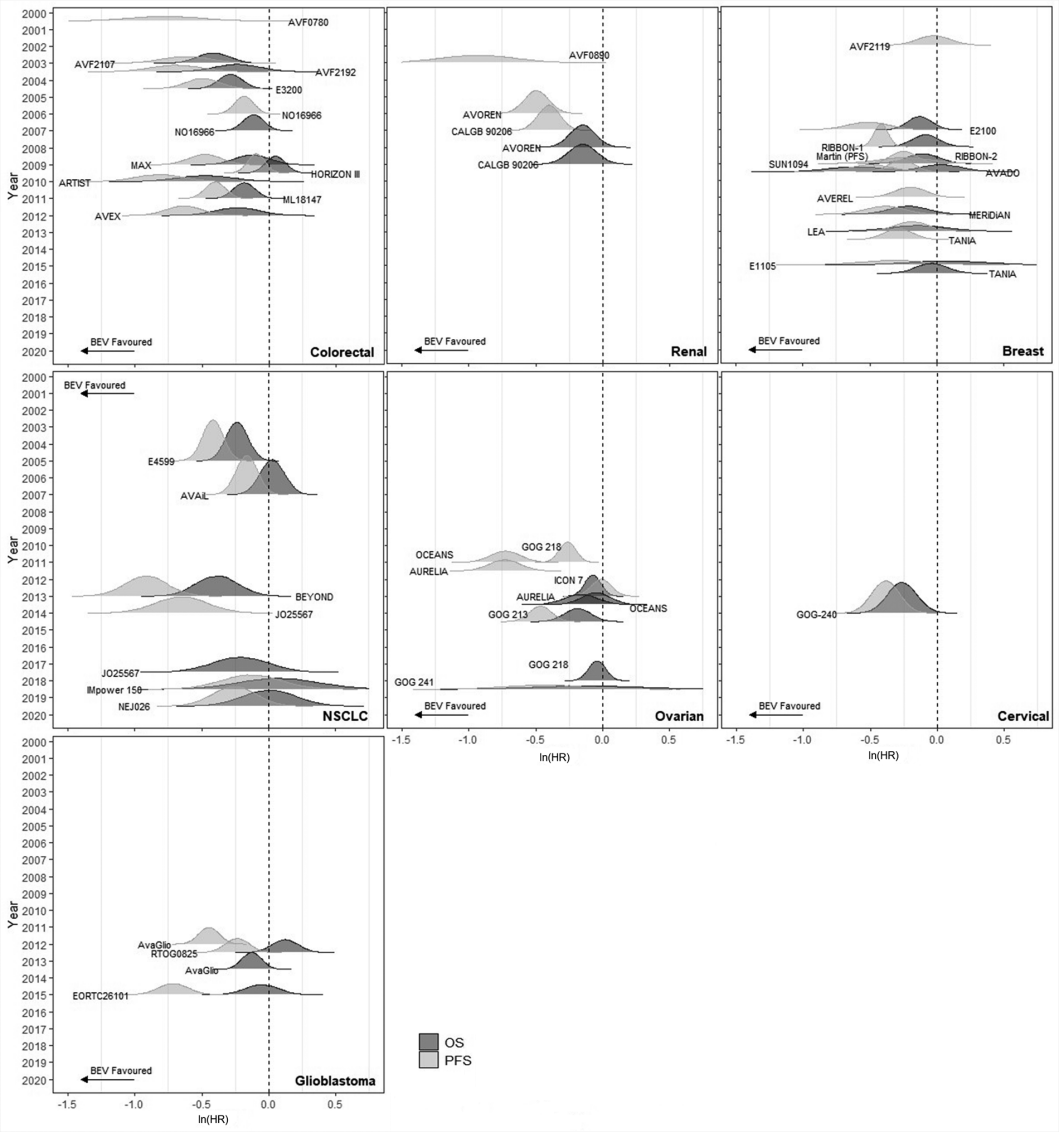
Ridgeline plots for all licensed indications for bevacizumab. BEV, bevacizumab; BRE, breast cancer; CER, cervical cancer; COL, colorectal cancer; GLIO, glioblastoma; HR, hazard ratio; NSCLC, non-small-cell lung cancer; OFTPP, ovarian, fallopian tube, and primary peritoneal cancer; OS, overall survival; PFS, progression-free survival; REN, renal cell carcinoma.

Ridgeline plots showing the density of the final reported ln(HR) for OS and PFS for each indication are presented in Figure 3 (data are available in Supplementary Tables S2 and S3). Curves overlap within and across indications for both outcomes, suggesting the RTE of bevacizumab is similar across indications, providing support for a multi-indication sharing model, although there is some heterogeneity between studies within indications. Plots for colorectal, breast, and ovarian cancers are potentially difficult to interpret as many trials were conducted around the same time. Supplementary Section D, Figure S5, includes ridgeline plots for all indications ordered by decreasing OS, which can be used to compare the final trial OS and PFS HRs within and between indications, without considering when the trials were conducted.

### Displaying Synthesis Results

**Figure 4:**
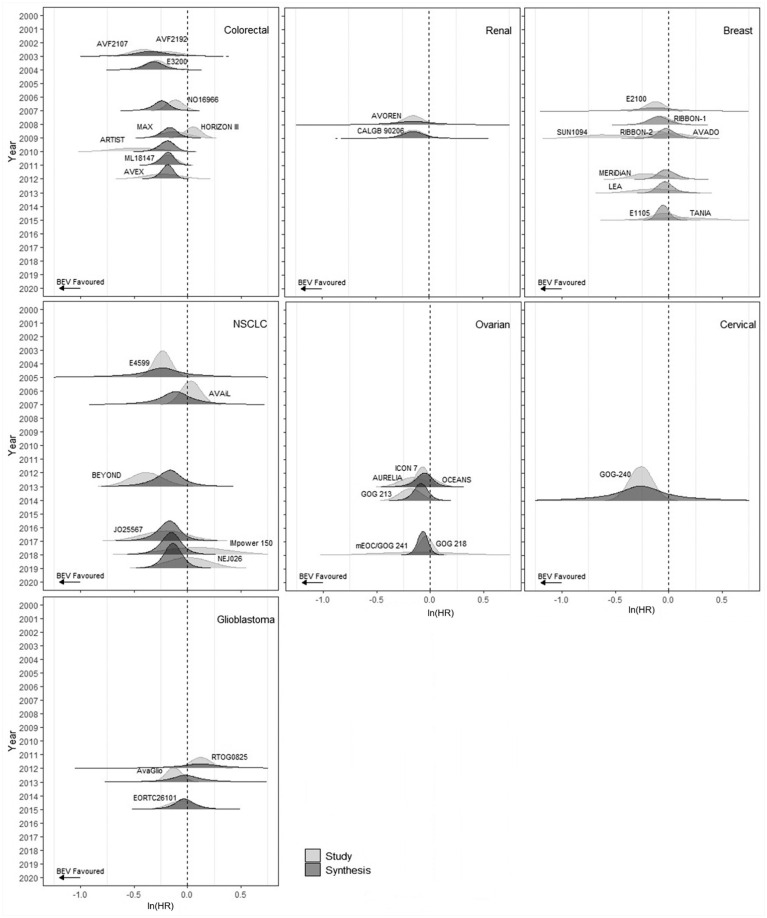
Ridgeline plots with cumulative independent parameter results for all licensed bevacizumab indications for overall survival. BEV, bevacizumab; BRE, breast cancer; CER, cervical cancer; COL, colorectal cancer; GLIO, glioblastoma; HR, hazard ratio; NSCLC, non-small-cell lung cancer; OFTPP, ovarian, fallopian tube and primary peritoneal cancer; REN, renal cell carcinoma.

Figure 4 shows the results of the cumulative meta-analysis using the IP model for each indication. As a random effects meta-analysis model is used, initially the meta-analysis results are more uncertain than the study results (influenced by the vague prior distribution assumed for the heterogeneity parameter), but as more evidence is added to the meta-analysis, the peaks of the synthesis curves get more pronounced, indicating an increase in the precision. Once 3 studies have been included in the cumulative analysis, the magnitude of the treatment effect (i.e., the position of the midpoint) stays largely consistent, and further increases in precision are small, due to the level of estimated within indication heterogeneity. Tables with all results, including estimated treatment effects, heterogeneity, and model fit statistics are presented in Supplementary Material Section B.

**Figure 5:**
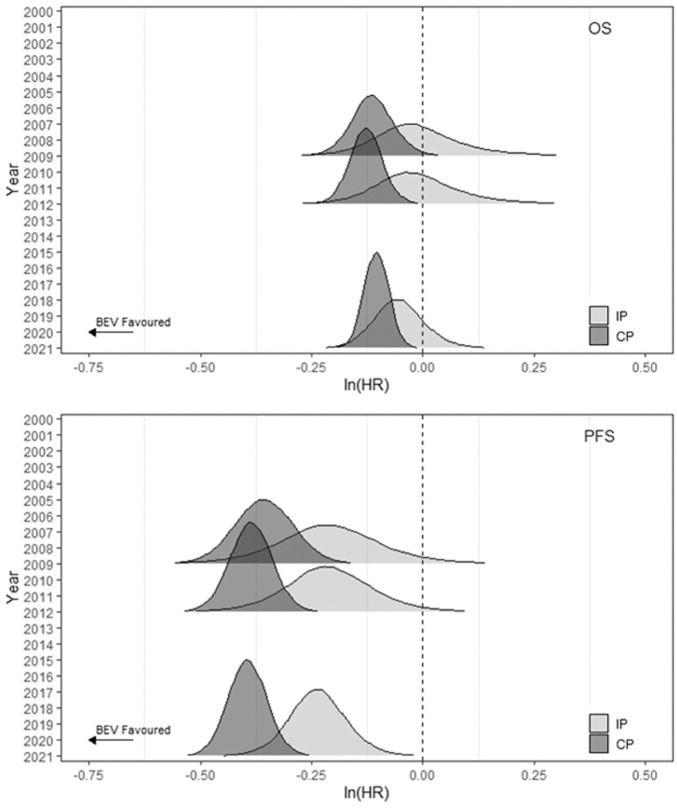
Ridgeline plots with results for breast cancer synthesis conducted at 3 prespecified time points using the IP and CP models for OS and PFS. BEV, bevacizumab; CP, common parameter, HR, hazard ratio; IP, independent parameter; OS, overall survival; PFS, progression-free survival.

When breast cancer is the target indication, assuming all studies for all indications available at each selected time point (2009, 2012, 2021) are appropriate for inclusion, ridgeline plots in Figure 5 display results from the IP and CP model at the 3 analysis time points for OS and PFS. Split-violin plots displaying the same results are reported in Supplementary Figure S6.

At time point 1 (2009), evidence for OS (PFS) from the 5 (6) breast cancer studies informs the IP model, but evidence from 10 (12) studies from 3 other indications are also included in the CP model. Thus, estimates from the CP model are more precise than for the IP model for both outcomes. Although densities estimated from the IP and CP models overlap, the peaks are not aligned, indicating some potential bias in the multi-indication synthesis results using the CP assumption (Figure 5) and suggesting that alternative sharing models should be assessed.^4,5^ At the second time point (2012), the increase in studies available for other indications to 18 for OS and 22 for PFS, and the further availability of PFS results for 1 breast cancer study, gives slightly more precise results than at time point 1, although the lack of overlap between distributions from both models remains. At the latest time point, the results for both models are more precise, and the OS results from the 2 models appear to be more aligned.

Results using ovarian cancer as the target indication (see Supplementary Section E) show good agreement between the IP and CP model results for both outcomes.

In both cases, estimates using the CP model for PFS are less precise than for OS, due to the larger estimated heterogeneity in PFS within breast and ovarian cancer studies (Supplementary Tables S8 and S9).

## Discussion

With an increase in the availability of multi-indication therapies, there is growing interest in approaches that make best use of the available evidence for HTA. Understanding the potentially complex evidence base is imperative to the assessment of the feasibility of multi-indication evidence synthesis and to evaluate the plausibility of results. The visualization methods discussed in this article can aid this understanding.

In our illustrative case study, while our aim was to identify as many RCTs comparing bevacizumab as possible, due to time and resource constraints the searches conducted were not comprehensive. We considered only licensed indications for bevacizumab as our aim was to judge similarity across comparable indications, and we expected relative effects in nonlicensed indications to be different from licensed indications as lack of efficacy could be a reason for no license. However, this should be discussed with topic experts on a case-by-case basis when considering which indications to include in the evidence synthesis. The evidence displays proposed can be used to help structure these discussions and make appropriate judgments.^5^

Bevacizumab was used as a case study due to its many licensed indications. However, it may have less complexity than more recent oncology drugs as the treatment effect is less likely to be modified by interactions with background treatments than other oncology drugs. This is because bevacizumab is deemed to administer its effect by its interaction on the stromal environment to the cancer as opposed to the tumor cells themselves, and there is likely to be more consistency between tumors in relation to this. Therefore, while clinical heterogeneity across studies was assessed to be small in this case study, this may not be the case for other multi-indication drugs.

In selecting the proposed evidence displays, we focused on features of oncology that can help assess RTE heterogeneity and the value of sharing of information across indications: evidence accumulation within and across indications, type of comparator, maturity, and precision/uncertainty. Plots can be extended to visualize other evidence features, including key events such as when a drug became available in clinical practice or causes for heterogeneity between and within indications such as subgroups, differences in study design, quality of studies, or statistical considerations such as nonproportional hazards, crossover adjustments, and stratification. The plots presented here can be tailored by, for example, using different symbols or colors in each plot to indicate different features or adding annotations to indicate the availability of data for different subgroups. Singh et al.^4^ included a representation of time points when bevacizumab was appraised by NICE in a timeline plot (presented as a supplementary figure). However, a tradeoff between presenting all relevant features and plot readability is required. Plots containing too much information can be cumbersome to interpret, which is a limitation of static evidence presentations. Here we preferred to keep the plot design simple and made judgments on key features to present. Making plots interactive so stakeholders can query the data further by, for example, clicking on regions of interest to expand or maximize the display, selecting particular studies for inclusion/exclusion in the display, or where the features being displayed could be turned on or off by the user would be a useful extension. In support of evidence synthesis, and as per current practice,^20,21,41^ our evidence displays highlight the indications/studies with the most precise evidence, which are expected to be more influential to the results of any analyses. These displays could, instead, be developed to visualize uncertainty, instead of precision, in which case, future research could integrate and perhaps expand on the broader research by MacEachren et al.^42^

For the visualization of RTEs, we used densities instead of the usual forest plot representation, an approach that was well received and understood by all clinical coauthors. Inconsistent reporting of trial summaries prevented effective visualization of maturity, which we consider a key evidence feature. The proposed plots extend naturally to the presentation of alternative measures of maturity or uncertainty that can be represented by a single number, although these may require digitizing KM curves, which carries an additional time burden.

The proposed plots can also be adapted to other contexts in which sharing of information may be beneficial, for example, when multiple drugs of the same class are used in the same indication or to assess whether sparse adverse event data may benefit from information sharing across indications or populations. Ridgeline plots in which the vertical axis indicates the evidence source (e.g., study name) and where direct (i.e., within class or indication/population) and indirect (from a different class or indication/population) evidence are presented using different shading or colors can help assess heterogeneity and consistency of RTEs between evidence sources. Similarly, ridgeline or split-violin plots could be adapted to display results from models including or excluding indirect evidence.

Other relevant outcomes used in HTA, such as response rate, may also need to be displayed to evaluate the heterogeneity and feasibility of information sharing, but they introduce additional challenges. The joint presentation of outcomes on different scales will require additional modifications to the plots.

An empirical assessment of whether the assumptions made for the CP model in our illustrative example were appropriate are beyond the scope of this work, but they are discussed in detail in Singh et al.^4^ using the same evidence base. The performance of different sharing models has also been evaluated in a simulation study.^5^ In the breast cancer example, the simple CP model with maximum sharing of evidence provided the most precise results but may add bias. The displays proposed here can be used as a starting point for discussion of key features and characteristics of the evidence with experts who can suggest studies/indications to include and candidate models for multi-indication synthesis or to exclude multi-indication synthesis as not appropriate or feasible. A discussion of the tradeoff between precision and bias is also needed before results can be considered suitable for decision making.^5^ This discussion can be facilitated by the plots proposed here, which can incorporate results from other fitted models. However, on their own, they cannot be used for a final decision on which model to use in decision making. As in any synthesis, expert opinion on the plausibility of assumptions and formal statistical checks for model fit should be considered.^4,5^

## Supplemental Material

sj-docx-1-mdm-10.1177_0272989X261430333 – Supplemental material for Visualization of Multi-indication Randomized Control Trial Evidence to Support Decision Making in Oncology: A Case Study on Bevacizumab Supplemental material, sj-docx-1-mdm-10.1177_0272989X261430333 for Visualization of Multi-indication Randomized Control Trial Evidence to Support Decision Making in Oncology: A Case Study on Bevacizumab by Sumayya Anwer, Janharpreet Singh, Sylwia Bujkiewicz, Anne Thomas, Richard Adams, Elizabeth Smyth, Pedro Saramago, Stephen Palmer, Marta O. Soares and Sofia Dias in Medical Decision Making
