# Supplementary material for: Visualization of Multi-indication Randomized Control Trial Evidence to Support Decision Making in Oncology: A Case Study on Bevacizumab
Source: Med Decis Making. 2026 Mar 31;46(6):716–29. doi: 10.1177/0272989X261430333 (PMC13346600; doi:10.1177/0272989X261430333)
Supplement: sj-docx-1-mdm-10.1177_0272989X261430333 – Supplemental material for Visualization of Multi-indication Randomized Control Trial Evidence to Support Decision Making in Oncology: A Case Study on Bevacizumab [file sj-docx-1-mdm-10.1177_0272989X261430333.docx]

**Supplementary Material**

Visualisation of multi-indication randomised control trial evidence to support decision-making in oncology: a case study on bevacizumab

**Table of Contents**

[A: Study identification and data extraction 2](#_Toc213684922)

[A-I: Identification of studies 2](#_Toc213684923)

[A-II: Data Extraction Details 5](#_Toc213684924)

[B: Statistical Methods and Results 10](#_Toc213684925)

[B-I: Description of statistical methods 10](#_Toc213684926)

[B-II: Results of the Synthesis Models 11](#_Toc213684927)

[B-III: Model Fit Statistics 15](#_Toc213684928)

[B-IV: Results of the Synthesis Models- Target Indication 19](#_Toc213684929)

[C: Key Features of Oncology Evidence 21](#_Toc213684930)

[C-I: Maturity 21](#_Toc213684931)

[C-II: Precision 23](#_Toc213684932)

[D: Additional Figures 25](#_Toc213684933)

[E: Additional figures for Ovarian Cancer as the target indication 34](#_Toc213684934)

[References 37](#_Toc213684935)

# A: Study identification and data extraction

## A-I: Identification of studies


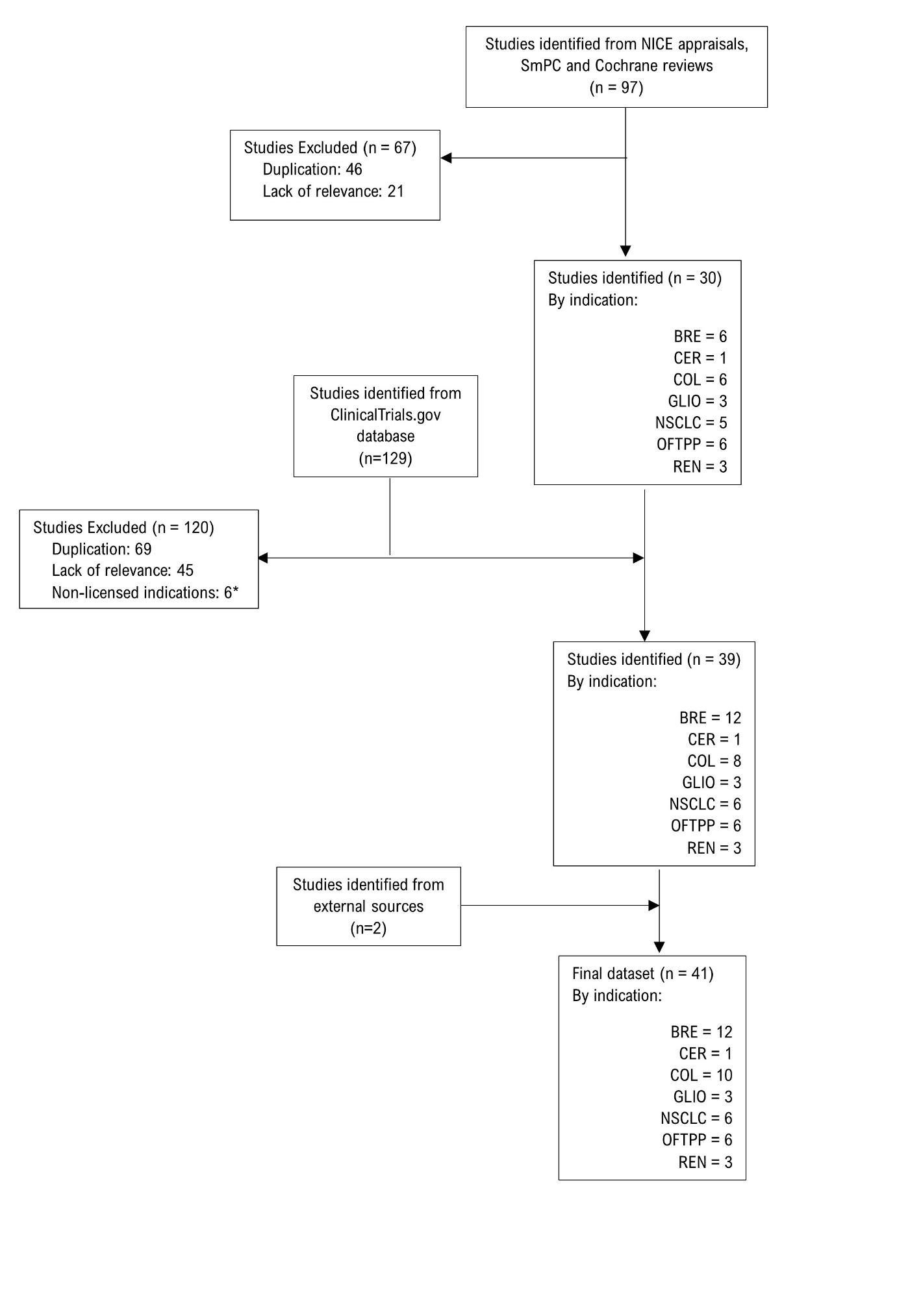
**Figure S1.** PRISMA diagram for the study search process

**Table S1.** Bevacizumab trials that were identified.

| **Study** | **Publications** | **Control** | **Comparator^‡^** |
| --- | --- | --- | --- |
| ***Breast Cancer*** | | | |
| AVF2119 | Miller (2005)^1^ | Capecitabine | Capecitabine + Bevacizumab |
| E2100 | Miller (2007)^2^ ; Cameron (2008)^3^ | Paclitaxel | Paclitaxel + Bevacizumab |
| RIBBON-1 | Robert (2011)^4^ | Capecitabine | Capecitabine + Bevacizumab |
|  |  | Taxane/ Anthracycline | Taxane/Anthracycline + Bevacizumab |
| RIBBON-2 | Brufsky (2011)^5^ | Chemotherapy | Chemotherapy + Bevacizumab |
| AVADO | Miles (2010)^6^; Miles (2013)^7^ | Docetaxel | Docetaxel + Bevacizumab (15mg/kg) |
| AVEREL | Gianni (2013)^8^ | Docetaxel +Trastuzumab | Docetaxel + Trastuzumab + Bevacizumab |
| SUN1094 | Robert (2011)^9^ | Paclitaxel + Sunitinib | Paclitaxel + Bevacizumab |
| Martin (2011) | Martin (2011)^10^ | Paclitaxel + Placebo | Paclitaxel+ Bevacizumab |
| LEA | Martin (2015)^11^ | Endocrine therapy | Endocrine therapy + Bevacizumab |
| E1105 | Artega (2012)^12^; Clinicaltrials.gov^13^ | Chemotherapy + Placebo | Chemotherapy + Bevacizumab |
| TANIA | Von Minckwitz (2014)^14^; Vrdoljak (2016)^15^ | Chemotherapy | Chemotherapy + Bevacizumab |
| MERiDiAN | Miles (2017)^16^ | Placebo + Paclitaxel | Bevacizumab + Paclitaxel |
| ***Cervical Cancer*** | | | |
| GOG 240 | Tewari (2014)^17^; Tewari (2017)^18^ | Chemotherapy | Chemotherapy + Bevacizumab |
| ***Colorectal Cancer*** | | | |
| AVF0780 | Kabbinavar (2003)^19^ | FL | FL + Bevacizumab (5 mg/kg) |
| AVF2192 | Kabbinavar (2005)^20^ | FL | FL + Bevacizumab |
| AVF2107 | Hurwitz (2004)^21^ | IFL + Placebo | IFL + Bevacizumab |
| E3200 | Giantonio (2007)^22^ | FOLFOX4 | FOLFOX4 + Bevacizumab |
| NO16966 | Saltz (2008)^23^; Cassidy (2011)^24^ | Chemotherapy | Chemotherapy + Bevacizumab |
| MAX | Tebbutt (2010)^25^ | Capecitabine | Capecitabine + Bevacizumab |
| ML18147 | Bennouna (2013)^26^; Kubicka (2013)^27^ | Chemotherapy | Chemotherapy + Bevacizumab |
| HORIZON III | Schmoll (2012)^28^ | mFOLFOX6 + Cediranib | mFOLFOX6 + Bevacizumab |
| AVEX | Cunningham (2013)^29^ | Capecitabine | Capecitabine + Bevacizumab |
| ARTIST | Guan (2011)^30^ | IFL | IFL + Bevacizumab |
| ***Glioblastoma*** | | | |
| RTOG0825 | Gilbert (2014)^31^ | Placebo | Placebo + Bevacizumab |
| AVAglio | Sandmann (2015)^32^ | Radiotherapy/ Temozolomide | Radiotherapy/Temozolomide + Bevacizumab |
| EORTC26101 | Wick (2017)^33^ | Lomustine | Lomustine + Bevacizumab |
| ***NSCLC*** | | | |
| E4599 | Sandler (2006)^34^ | Carboplatin + Paclitaxel | Carboplatin + Paclitaxel + Bevacizumab |
| AVAiL | Reck (2009)^35^; Reck (2010)^36^ | Cisplatin + Gemcitabine + Placebo | Cisplatin + Gemcitabine + Bevacizumab (15 mg/kg) |
| JO25567 | Seto (2014)^37^; Yamamoto (2021)^38^ | Erlotinib | Erlotinib + Bevacizumab |
| BEYOND | Zhou (2015)^39^ | Carboplatin + Paclitaxel + Placebo | Carboplatin + Paclitaxel + Bevacizumab |
| IMpower150 | Reck (2019)^40^; Socinski (2021)^41^ | Carboplatin + Paclitaxel + Atezolizumab | Carboplatin + Paclitaxel + Bevacizumab |
| NEJ026 | Saito (2019)^42^; Kawashima (2022)^43^ | Erlotinib | Erlotinib + Bevacizumab |
| ***Ovarian, fallopian tube, and primary peritoneal cancer*** | | | |
| GOG218 | Burger (2011)^44^; Tewari (2019)^45^ | Carboplatin + Paclitaxel + Placebo | Carboplatin + Paclitaxel + Bevacizumab |
| ICON7 | Perren (2011)^46^; Oza (2015)^47^ | Carboplatin + Paclitaxel | Carboplatin + Paclitaxel + Bevacizumab |
| OCEANS | Aghajanian (2012)^48^; Aghajanian (2015)^49^ | Gemcitabine + Carboplatin + Placebo | Gemcitabine + Carboplatin + Bevacizumab |
| GOG213 | Coleman (2017)^50^ | Carboplatin + Paclitaxel | Carboplatin + Paclitaxel + Bevacizumab |
| AURELIA | Pujade-Lauraine (2014)^51^; Bamias (2017)^52^ | Chemotherapy | Chemotherapy + Bevacizumab |
| mEOC/GOG241 | Gore (2019)^53^ | Carboplatin + Paclitaxel | Carboplatin + Paclitaxel + Bevacizumab |
|  |  | Oxaliplatin + Capecitabine | Oxaliplatin + Capecitabine + Bevacizumab |
| ***Renal cell carcinoma*** | | | |
| AVF0890 | Yang (2003)^54^ | Placebo | Bevacizumab (10 mg/kg) |
| CALGB-90206 | Rini (2008)^55^; Rini (2010)^56^ | Interferon | Interferon + Bevacizumab |
| AVOREN | Escuidier (2007)^57^; Escuidier (2010)^58^ | Interferon + Placebo | Interferon + Bevacizumab |
| ***Gastrointestinal cancer^†^*** | | | |
| AVATAR | Shen (2015)^59^ | Capecitabine + Cisplatin + Placebo | Capecitabine + Cisplatin + Bevacizumab |
| AVAGAST | Ohtsu (2011)^60^ | Capecitabine + Cisplatin + Placebo | Capecitabine + Cisplatin + Bevacizumab |
| ***Lymphoma^†^*** | | | |
| MAIN | Seymour (2014)^61^ | R-CHOP* | R-CHOP + Bevacizumab |
| ***Urothelial cancer^†^*** | | | |
| CALGB-90601 | Rosenberg (2021)^62^ | Cisplatin + Gemcitabine + Placebo | Cisplatin + Gemcitabine + Bevacizumab |
| ***Prostate cancer^†^*** | | | |
| CALGB-90401 | Kelly (2012)^63^ | Docetaxel + Prednisone | Docetaxel + Prednisone + Bevacizumab |
| ***Uterine cancer^†^*** | | | |
| GOG250 | Hensley (2015)^64^ | Gemcitabine + Docetaxel + Placebo | Gemcitabine + Docetaxel + Bevacizumab |

***** R-CHOP consists of Rituximab, Cyclophosphamide, Doxorubicin, Vincristine, and Prednisone. † These cancer indications were not included in the work conducted in this paper.
‡Where a dose is specified for bevacizumab, that was the trial arm that data were extracted from when a trial looked at multiple doses of bevacizumab. **Treatment abbreviations:** FL- leucovorin and fluorouracil; IFL-irinotecan, leucovorin and fluorouracil; FOLFOX4- oxaliplatin, leucovorin, and 5- fluorouracil; mFOLFOX6- modified FOLFOX6; R-CHOP-rituximab, cyclophosphamide, doxorubicin, vincristine and prednisone.

## A-II: Data Extraction Details

For all included studies we extracted relevant trial characteristics as well as outcome data.

Trial characteristics:

We extracted the trial location and number of centres in the trials, details on treatment regimens (including doses, frequency, and duration of treatment). For trials where different doses of bevacizumab were compared to each other as well as a comparator treatment, we only extracted evidence from the treatment arm that used the dose licensed for that particular indication. We also extracted the length of follow-up in each trial.

Patient characteristics:

We extracted the patient demographics including age, sex, ECOG performance score, and prior treatment history, noted where subgroup analysis had been conducted on different patient characteristics.

Outcome Data:

We extracted outcome data for overall survival (OS), progression-free (PFS), and response. For OS and PFS, we extracted the reported hazard ratio (HR) and 95% CI and, where reported, the number of participants who experienced an event (i.e. progression or death). For PFS we also extracted how progression was assessed and where trials reported more than one method, Independent Reviewer Committee/Facility (IRC/IRF) was preferred over investigator assessment.

For response, we recorded the overall response rates (ORRs), and the number of patients who experienced complete or partial response (CR or PR); however we did not explore response as an outcome in our visualisations.

The extracted data that used in the figures and in all analyses that were conducted are reported in Table S2 (for OS) and Table S3 (for PFS)

**Table S2**. Extracted data for OS

| **Trial** | **Publication** | **Cut-off date^†^** | **Randomised Patients** | | **Number of Events** | | **Hazard Ratio**  **(95% CI)** |
| --- | --- | --- | --- | --- | --- | --- | --- |
|  |  |  | **Control** | **Comparator** | **Control** | **Comparator** |  |
| ***Colorectal cancer*** | | | | | | | |
| AVF2192 | Kabbinavar (2005) | 01/09/2003 | 105 | 104 | NR | NR | 0.79 (0.56, 1.10) |
| AVF2107 | Hurwitz (2004) | 01/04/2003 | 411 | 402 | NR | NR | 066 (0.52, 0.84) |
| E3200 | Giantonio (2007) | 01/05/2004 | 291 | 286 | NR | NR | 0.75 (0.63, 1.89) |
| NO16966 | Saltz (2008) | 01/02/2007 | 701 | 699 | NR | NR | 0.89 (0.76, 1.03) |
| MAX | Tebbutt (2010) | 27/02/2009 | 156 | 157 | NR | NR | 0.88 (0.68, 1.13) |
| ML18147 | Bennouna (2013) | 01/05/2011 | 411 | 409 | NR | NR | 0.83 (0.71, 0.97) |
| HORIZON-III | Schmoll (2012) | 15/11/2009 | 709 | 713 | 239 | 247 | 1.05 (0.91, 1.22) |
| AVEX | Cunningham (2013) | 19/01/2012 | 140 | 140 | NR | NR | 0.79 (0.57, 1.09) |
| ARTIST | Guan (2011) | 01/12/2010 | 64 | 139 | NR | NR | 0.62 (0.41, 0.95) |
| ***Renal cell carcinoma*** | | | | | | | |
| CALGB-90206 | Rini (2010) | 01/03/2009 | 363 | 369 | NR | NR | 0.86 (0.73, 1.10) |
| AVOREN | Escudier (2007) | 01/08/2006 | 322 | 327 | 137 | 114 | 0.75 (0.58, 0.97) |
|  | Escudier (2010) | 01/09/2008 | 322 | 327 | 224 | 220 | 0.86 (0.72, 1.04) |
| ***Breast cancer*** | | | | | | | |
| E2100 | Cameron (2008) | 01/10/2006 | 354 | 368 | NR | NR | 0.87 (0.72, 1.05) |
|  | Miller (2007) | 01/06/2007 | 354 | 368 | NR | NR | 0.88 (0.74, 1.05) |
| RIBBON-1^‡^ | Robert (2011) | 01/07/2008 | 206 | 409 | NR | NR | 0.85 (0.63, 1.14) |
|  |  |  | 207 | 415 | NR | NR | 1.03 (0.77, 1.38) |
| RIBBON-2 | Brufsky (2011) | 01/03/2009 | 255 | 459 | 109 | 206 | 0.90 (0.71, 1.33) |
| AVADO | Miles (2010) & (2013) | 01/04/2009 | 241 | 247 | 133 | 131 | 1.03 (0.70, 1.33) |
| SUN1094 | Robert (2011) | 01/06/2009 | 242 | 243 | 52 | 32 | 0.55 (0.35, 0.86) |
| LEA | Martin (2015) | 01/12/2013 | 184 | 190 | 46 | 47 | 0.87 (0.58, 1.32) |
| E1105 | Clinical Trials Results | 01/10/2015 | 48 | 48 | NR | NR | 1.09 (0.61, 1.97) |
| TANIA | Vrdoljak (2016) | 30/04/2015 | 247 | 247 | 156 | 163 | 0.96 (0.76, 1.21) |
| MERiDiAN | Miles (2017) | 30/11/2014 | 233 | 238 | 105 | 91 | 0.81 (0.61, 1.08) |
| ***NSCLC*** | | | | | | | |
| E4599 | Sandler (2005) | 01/10/2005 | 444 | 434 | 344 | 305 | 0.79 (0.67, 0.92) |
| AVAiL | Reck (2010) | 01/11/2007 | 347 | 351 | 240 | 242 | 1.03 (0.86, 1.23) |
| J025567 | Yamamoto (2021) | 01/10/2017 | 77 | 75 | NR | NR | 0.81 (0.53, 1.23) |
| BEYOND | Zhou (2015) | 27/01/2013 | 138 | 138 | NR | NR | 0.68 (0.50, 0.93) |
| IMpower150 | Reck (2019) | 01/01/2018 | 402 | 400 | NR | NR | 1.08 (0.60, 1.96) |
| NEJ026 | Kawashima (2022) | 01/11/2019 | 114 | 114 | NR | NR | 1.01 (0.68, 1.49) |
| ***Ovarian, fallopian tube, and primary peritoneal cancer*** | | | | | | | |
| GOG218 | Burger (2011) | 01/02/2010 | 625 | 623 | 156 | 138 | 0.92 (0.73, 1.15) |
|  | Burger (2011) | 01/08/2011 | 625 | 623 | 298 | 269 | 0.89 (0.75, 1.04) |
|  | Tewari (2019) | 01/01/2018 | 625 | 623 | NR | NR | 0.96 (0.85, 1.09) |
| ICON7 | Perren (2011) | 01/02/2010 | 764 | 764 | 130 | 111 | 0.81 (0.63, 1.04) |
|  | Perren (2011) | 01/11/2010 | 764 | 764 | 200 | 178 | 0.85 (0.69, 1.04) |
|  | Oza (2015) | 01/03/2013 | 764 | 764 | 352 | 362 | 0.99 (0.85, 1.14) |
| OCEANS | Aghajanian (2012) | 01/09/2010 | 242 | 242 | NR | NR | 0.75 (0.54, 1.05) |
|  | Aghajanian (2012) | 01/08/2011 | 242 | 242 | NR | NR | 1.03 (0.79, 1.33) |
|  | Aghajanian (2015) | 01/07/2013 | 242 | 242 | NR | NR | 0.95 (0.77, 1.18) |
| GOG213 | Coleman (2017) | 01/11/2014 | 337 | 337 | 214 | 201 | 0.83 (0.68, 1.01) |
| AURELIA | Pujade-Lauraine (2014) | 01/01/2013 | 182 | 179 | 136 | 128 | 0.85 (0.66, 1.08) |
| mEOC/GOG241^‡^ | Gore (2019) | 01/02/2018 | 13 | 11 | NR | NR | 1.47 (0.56, 3.84) |
|  |  |  | 13 | 13 | NR | NR | 0.77 (0.29, 2.03) |
| ***Cervical cancer*** | | | | | | | |
| GOG240 | Tewari (2014) | 01/03/2012 | 225 | 227 | 140 | 131 | 0.71 (0.54, 0.95) |
|  | Tewari (2017) | 01/03/2014 | 225 | 227 | 175 | 173 | 0.77 (0.62, 0.95) |
| ***Glioblastoma*** | | | | | | | |
| RTOG0825 | Glibert (2014) | 01/10/2015 | 317 | 320 | 198 | 215 | 1.13 (0.93, 1.37) |
| AvaGlio | Chinot (2014) | 01/02/2013 | 463 | 458 | NR | NR | 0.88 (0.76, 1.02) |
| EORTC26101 | Wick (2017) | 01/10/2015 | 149 | 288 | 113 | 216 | 0.95 (0.74, 1.21) |

† Where studies only reported month and year for the data cut-off, we assumed that this was the first of the month. ‡Where studies reported multiple two-arm (chemotherapy vs. chemotherapy +bevacizumab) comparisons, both were included as long as there was no overlap in patients.
**Abbreviations:** CI, confidence interval; NR, not reported; NSCLC, non-small cell lung cancer

**Table S3.** Extracted data for PFS

| **Trial** | **Publication** | **Cut-off date^†^** | **Assessment Method** | **Randomised Patients** | | **Number of Events** | | **Hazard Ratio**  **(95% CI)** |
| --- | --- | --- | --- | --- | --- | --- | --- | --- |
|  |  |  |  | **Control** | **Comparator** | **Control** | **Comparator** |  |
| ***Colorectal cancer*** | | | | | | | | |
| AVF0780 | Kabbinavar (2003) | 01/10/2000 | IRF | 36 | 35 | 26 | 22 | 0.46 (0.27, 0.79) |
| AVF2192 | Kabbinavar (2005) | 01/09/2003 | IRF | 105 | 104 | NR | NR | 0.50 (0.34, 0.73) |
| AVF2107 | Hurwitz (2004) | 01/04/2003 | IRC | 411 | 402 | NR | NR | 0.54 (0.37, 0.78) |
| E3200 | Giantonio (2007) | 01/05/2004 | INV | 291 | 286 | NR | NR | 0.61 (0.48, 078) |
| NO16966 | Saltz (2008) | 01/02/2006 | INV | 701 | 699 | NR | NR | 0.83 (0.72, 0.95) |
| MAX | Tebbutt (2010) | 27/02/2009 | NR | 156 | 157 | NR | NR | 0.62 (0.49, 0.79) |
| ML18147 | Bennouna (2013) | 01/05/2011 | INV | 411 | 409 | NR | NR | 0.67 (0.58, 0.78) |
| HORIZON-III | Schmoll (2012) | 15/11/2009 | NR | 709 | 713 | 471 | 453 | 0.91 (0.80, 1.03) |
| AVEX | Cunningham (2013) | 19/01/2012 | NR | 140 | 140 | NR | NR | 0.53 (0.41, 0.69) |
| ARTIST | Guan (2011) | 01/12/2010 | INV | 64 | 139 | NR | NR | 0.44 (0.31, 0.63) |
| ***Renal cell carcinoma*** | | | | | | | | |
| AVF0890 | Yang (2003) | 01/02/2003 | NR | 40 | 39 | NR | NR | 0.39 (0.23, 0.68) |
| CALGB-90206 | Rini (2008) | 01/10/2007 | INV | 363 | 369 | NR | NR | 0.67 (0.57, 0.79) |
| AVOREN | Escudier (2007) | 01/08/2006 | INV | 322 | 327 | 275 | 230 | 0.61 (0.51, 0.73) |
| ***Breast Cancer*** | | | | | | | | |
| AVF2119 | Miller (2005) | 01/06/2002 | IRC | 230 | 232 | NR | NR | 0.98 (0.77, 1.25) |
| E2100 | Cameron (2008) | 01/02/2005 | INV | 354 | 368 | 244 | 201 | 0.42 (0.34, 0.52) |
|  | Cameron (2008) | 01/04/2005 | IRC | 354 | 368 | 184 | 173 | 0.48 (0.33, 0.69) |
|  | Miller (2007) | 01/06/2007 | NR | 326 | 347 | 308 | 316 | 0.60 (0.44, 0.81) |
| RIBBON-1^‡^ | Robert (2011) | 01/07/2008 | IRC | 206 | 409 | NR | NR | 0.69 (0.56, 0.84) |
|  |  |  |  | 207 | 415 | NR | NR | 0.64 (0.52, 0.80) |
| RIBBON-2 | Brufsky (2011) | 01/03/2009 | INV | 255 | 459 | 184 | 372 | 0.78 (0.64, 0.93) |
| AVADO | Miles (2013) | 01/10/2007 | INV | 241 | 247 | NR | NR | 0.61 (0.48, 0.78) |
|  | Miles (2010) | 01/04/2009 | INV | 241 | 247 | 219 | 220 | 0.77 (0.64, 0.93) |
| AVEREL | Gianni (2013) | 30/06/2011 | INV | 208 | 216 | 154 | 153 | 0.82 (0.65, 1.02) |
| SUN1094 | Robert (2011) | 01/06/2009 | NR | 242 | 243 | 89 | 70 | 0.61 (0.44, 0.85) |
| Martin (2011) | Martin (2011) | 01/05/2009 | IRC | 94 | 97 | 15 | 9 | 0.79 (0.53, 1.17) |
| LEA | Martin (2015) | 01/12/2013 | NR | 184 | 190 | 135 | 128 | 0.83 (0.65, 1.06) |
| E1105 | Clinical Trials | 01/10/2015 | NR | 48 | 48 | NR | NR | 0.73 (0.43, 1.23) |
| TANIA | von Minckwitz (2014) | 20/12/2013 | INV | 247 | 247 | 203 | 204 | 0.75 (0.61, 0.93) |
| MERiDiAN | Miles (2017) | 30/11/2014 | INV | 233 | 238 | 168 | 152 | 0.68 (0.51, 0.91) |
| ***Non-small cell lung cancer*** | | | | | | | | |
| E4599 | Sandler (2005) | 01/10/2005 | NR | 444 | 434 | 405 | 374 | 0.66 (0.57, 0.77) |
| AVAiL | Reck (2019) | 01/10/2006 | INV | 347 | 351 | NR | NR | 0.82 (0.68, 0.98) |
|  | Reck (2010) | 01/11/2007 | INV | 347 | 351 | NR | NR | 0.85 (0.73, 1.00) |
| J025567 | Seto (2014) | 01/06/2013 | IRC | 77 | 75 | 57 | 46 | 0.54 (0.36, 0.79) |
|  | Yamamoto (2021) | 01/03/2014 | INV | 77 | 75 | NR | NR | 0.52 (0.35, 0.76) |
| BEYOND | Zhou (2015) | 27/01/2013 | INV | 138 | 138 | NR | NR | 0.40 (0.29, 0.54) |
| IMpower150 | Reck (2019) | 01/01/2018 | INV | 402 | 400 | NR | NR | 0.88 (0.56, 1.37) |
| NEJ026 | Saito (2019) | 01/09/2017 | IRC | 114 | 114 | NR | NR | 0.61 (0.42, 0.88) |
|  | Kawashima (2022) | 01/11/2019 | INV | 114 | 114 | NR | NR | 0.77 (0.56, 1.07) |
| ***Ovarian, fallopian tube, and primary peritoneal cancer*** | | | | | | | | |
| GOG218 | Burger (2011) | 01/02/2010 | NR | 625 | 623 | NR | NR | 0.72 (0.63, 0.82) |
|  | Burger (2011) | 01/08/2011 | NR | 625 | 623 | NR | NR | 0.77 (0.68, 0.87) |
| ICON7 | Perren (2011) | 01/02/2010 | INV | 764 | 764 | 392 | 367 | 0.81 (0.70, 0.94) |
|  | Perren (2011) | 01/11/2010 | INV | 764 | 764 | 392 | 367 | 0.87 (0.77, 0.99) |
|  | Oza (2015) | 01/03/2013 | INV | 764 | 764 | 526 | 554 | 0.93 (0.83, 1.05) |
| OCEANS | Aghajanian (2012) | 01/08/2011 | INV | 242 | 242 | 187 | 151 | 0.48 (0.39, 0.61) |
| GOG213 | Coleman (2017) | 01/11/2014 | INV | 337 | 337 | NR | NR | 0.63 (0.53, 0.74) |
| AURELIA | Pujade-Lauraine (2014) | 01/01/2013 | INV | 182 | 179 | 166 | 135 | 0.48 (0.38, 0.60) |
| mEOC/GOG241^‡^ | Gore (2019) | 01/02/2018 | NR | 13 | 11 | NR | NR | 1.12 (0.45, 2.80) |
|  |  |  |  | 13 | 13 | NR | NR | 0.55 (0.21, 1.45) |
| ***Cervical cancer*** | | | | | | | | |
| GOG240 | Tewari (2014) | 01/03/2012 | NR | 225 | 227 | 184 | 183 | 0.67 (0.54, 0.82) |
|  | Tewari (2017) | 01/03/2014 | NR | 225 | 227 | 206 | 199 | 0.68 (0.56, 0.84) |
| ***Glioblastoma*** | | | | | | | | |
| RTOG0825 | Gilbert (2014) | 01/12/2012 | NR | 317 | 320 | 256 | 256 | 0.79 (0.66, 0.94) |
| AvaGlio | Chinot (2014) | 01/03/2012 | IRC | 463 | 458 | 387 | 354 | 0.64 (0.55, 0.74) |
| EORTC26101 | Wick (2017) | 01/10/2015 | IRC | 149 | 288 | 143 | 260 | 0.49 (0.39, 0.61) |

† Where studies only reported month and year for the data cut-off, we assumed that this was the first of the month. ‡ Where studies reported multiple two-arm (chemotherapy vs. chemotherapy +bevacizumab) comparisons, both were included as long as there was no overlap in patients.
**Abbreviations:** AM, assessment method; CI, confidence interval; IRC, independent review committee; IRF, independent review facility; INV, investigator assessment; NR, not reported, NSCLC, non-small cell lung cancer.

# B: Statistical Methods and Results

## B-I: Description of statistical methods

The random-effects meta-analysis normal-normal hierarchical model^65^ is used for within-indication meta-analysis. The relative treatment effect (for example the ln(HR)), *Y_ij_*, is assumed to follow a normal distribution:

$$Y_{ij}\sim N(\delta_{ij},\sigma_{ij}^{2})$$

where $\delta_{ij}$is the mean treatment effect and $\sigma_{ij}^{2}$ is the associated standard error for study *i* within indication *j*. The mean treatment effect, $\delta_{ij}$ is assumed to be exchangeable across studies within each indication:

$$\delta_{ij}\sim N(d_{j},\tau_{j}^{2})$$

where $d_{j}$ is the pooled treatment effect and $\tau_{j}$ is the between-study standard deviation, i.e. the within-indication (heterogeneity). A weakly-informative half-normal prior distribution is placed on the between-study standard deviation for each indication:^66^

$$\tau_{j}\sim|N(0,0.5^{2})|$$

Assumptions on the degree of information sharing across indications differed for the two models we explored here:

1. Independent parameter (IP) meta-analysis model

As there is no evidence sharing across indications, a vague normal prior distribution, $d_{j}\sim N(0,1000)$ is used for the pooled, indication-specific relative treatment effect, $d_{j}$, for each indication *j*.

1. Common parameter (CP) meta-analysis model

In this model there is complete sharing of information, $d_{j}$ is replaced by a common parameter, $d$, which pools treatment effects across all indications. This common/pooled RTE is assigned a vague normal prior distribution, $d\sim N(0,1000)$.

## B-II: Results of the Synthesis Models

**Table S4**. Synthesis results for overall survival. *Note: The treatment effect estimate is reported as the HR and corresponding 95% credible interval on the log-scale.*

| **Time Point** |  | **CPMA Model** | **IPMA Model** |
| --- | --- | --- | --- |
| ***Colorectal Cancer*** |  |  |  |
| 31/12/2003  2 datapoints (2 in colorectal cancer) | Treatment Effect Estimate | -0.341 (-0.906, 0.253) | -0.341 (-0.906, 0.253) |
|  | Within-Indication SD | 0.207 (0.009, 0.889) | 0.207 (0.009, 0.889) |
| 31/12/2004  3 datapoints (3 in colorectal cancer) | Treatment Effect Estimate | -0.318 (-0.630, -0.002) | -0.318 (-0.630, -0.002) |
|  | Within-Indication SD | 0.117 (0.006, 0.627) | 0.117 (0.006, 0.627) |
| 31/12/2007  7 datapoints (4 in colorectal cancer) | Treatment Effect Estimate | -0.198 (-0.348, -0.041) | -0.248 (-0.502, -0.019) |
|  | Within-Indication SD | 0.120 (0.007, 0.463) | 0.130 (0.009, 0.527) |
| 31/12/2009  16 datapoints (6 in colorectal cancer) | Treatment Effect Estimate | -0.114 (-0.208, -0.019) | -0.171 (-0.374, 0.010) |
|  | Within-Indication SD | 0.151 (0.029, 0.392) | 0.161 (0.039, 0.435) |
| 31/12/2010  17 datapoints (7 in colorectal cancer) | Treatment Effect Estimate | -0.123 (-0.221, -0.026) | -0.196 (-0.392, -0.030) |
|  | Within-Indication SD | 0.157 (0.039, 0.391) | 0.164 (0.048, 0.415) |
| 31/12/2011  18 datapoints (8 in colorectal cancer) | Treatment Effect Estimate | -0.131 (-0.219, -0.041) | -0.190 (-0.350, -0.056) |
|  | Within-Indication SD | 0.133 (0.026, 0.330) | 0.138 (0.031, 0.340) |
| 31/12/2012  20 datapoints (9 in colorectal cancer) | Treatment Effect Estimate | -0.128 (-0.213, -0.038) | -0.191 (-0.332, -0.073) |
|  | Within-Indication SD | 0.125 (0.021, 0.307) | 0.127 (0.024, 0.300) |
| ***Renal cell carcinoma*** | | | |
| 31/12/2008  10 datapoints (1 in RCC) | Treatment Effect Estimate | -0.166 (-0.285, -0.042) | -0.150 (-1.251, 0.939) |
|  | Within-Indication SD | 0.161 (0.007, 0.840) | 0.337 (0.016, 1.121) |
| 31/12/2009  16 datapoints (2 in RCC) | Treatment Effect Estimate | -0.114 (-0.208, -0.018) | -0.151 (-0.643, 0.340) |
|  | Within-Indication SD | 0.094 (0.004, 0.574) | 0.152 (0.006, 0.823) |
| ***Breast cancer*** | | | |
| 31/12/2007  7 datapoints (1 in breast cancer) | Treatment Effect Estimate | -0.197 (-0.348, -0.041) | -0.128 (-1.235, 0.992) |
|  | Within-Indication SD | 0.183 (0.008, 0.862) | 0.339 (0.016, 1.133) |
| 31/12/2008  11 datapoints (3 in breast cancer) | Treatment Effect Estimate | -0.166 (-0.285, -0.043) | -0.095 (-0.411, 0.230) |
|  | Within-Indication SD | 0.102 (0.005, 0.510) | 0.122 (0.006, 0.646) |
| 31/12/2009  16 datapoints (6 in breast cancer) | Treatment Effect Estimate | -0.113 (-0.209, -0.015) | -0.019 (-0.208, 0.240) |
|  | Within-Indication SD | 0.133 (0.006, 0.484) | 0.152 (0.009, 0.519) |
| 31/12/2012  20 datapoints (6 in breast cancer) | Treatment Effect Estimate | -0.128 (-0.215, -0.040) | -0.018 (-0.211, 0.242) |
|  | Within-Indication SD | 0.142 (0.007, 0.502) | 0.152 (0.008, 0.518) |
| 31/12/2013  26 datapoints (7 in breast cancer) | Treatment Effect Estimate | -0.115 (-0.188, -0.043) | -0.035 (-0.195, 0.173) |
|  | Within-Indication SD | 0.106 (0.005, 0.402) | 0.123 (0.006, 0.435) |
| 31/12/2015  32 datapoints (10 in breast cancer) | Treatment Effect Estimate | -0.113 (-0.174, -0.054) | -0.055 (-0.168, 0.076) |
|  | Within-Indication SD | 0.068 (0.003, 0.270) | 0.073 (0.003, 0.280) |
| ***Non-small cell lung cancer*** | | | |
| 31/12/2005  4 datapoints (1 in NSCLC) | Treatment Effect Estimate | -0.296 (-0.501, -0.084) | -0.235 (-1.335, 0.856) |
|  | Within-Indication SD | 0.188 (0.008, 0.872) | 0.335 (0.015, 1.120) |
| 31/12/2007  7 datapoints (2 in NSCLC) | Treatment Effect Estimate | -0.197 (-0.348, -0.041) | -0.108 (-0.731, 0.520) |
|  | Within-Indication SD | 0.208 (0.020, 0.758) | 0.269 (0.028, 0.945) |
| 31/12/2013  26 datapoints (3 in NSCLC) | Treatment Effect Estimate | -0.116 (-0.187, -0.043) | -0.174 (-0.625, 0.243) |
|  | Within-Indication SD | 0.173 (0.016, 0.609) | 0.235 (0.028, 0.796) |
| 31/12/2017  33 datapoints (4 in NSCLC) | Treatment Effect Estimate | -0.116 (-0.176, -0.056) | -0.177 (-0.501, 0.114) |
|  | Within-Indication SD | 0.148 (0.012, 0.497) | 0.185 (0.018, 0.626) |
| 31/12/2018  37 datapoints (5 in NSCLC) | Treatment Effect Estimate | -0.104 (-0.161, -0.048) | -0.156 (-0.412, 0.103) |
|  | Within-Indication SD | 0.139 (0.011, 0.446) | 0.168 (0.016, 0.540) |
| 31/12/2019  38 datapoints (6 in NSCLC) | Treatment Effect Estimate | -0.104 (-0.161, -0.048) | -0.137 (-0.346, 0.080) |
|  | Within-Indication SD | 0.124 (0.008, 0.381) | 0.149 (0.014, 0.456) |
| ***Ovarian, fallopian tube and primary peritoneal cancer*** | | | |
| 31/12/2013  26 datapoints (3 in OFTPP cancer) | Treatment Effect Estimate | -0.115 (-0.187, -0.042) | -0.058 (-0.353, 0.210) |
|  | Within-Indication SD | 0.088 (0.004, 0.427) | 0.101 (0.004, 0.598) |
| 31/12/2014  29 datapoints (4 in OFTPP cancer) | Treatment Effect Estimate | -0.124 (-0.189, -0.060) | -0.090 (-0.282, 0.087) |
|  | Within-Indication SD | 0.075 (0.004, 0.321) | 0.084 (0.003, 0.418) |
| 31/12/2018  37 datapoints (7 in OFTPP cancer) | Treatment Effect Estimate | -0.105 (-0.161, -0.050) | -0.070 (-0.190, 0.042) |
|  | Within-Indication SD | 0.056 (0.003, 0.221) | 0.056 (0.003, 0.255) |
| ***Cervical cancer*** | | | |
| 31/12/2014  29 datapoints (1 in cervical cancer) | Treatment Effect Estimate | -0.125 (-0.189, -0.059) | -0.262 (-1.367, 0.842) |
|  | Within-Indication SD | 0.214 (0.010, 0.899) | 0.334 (0.016, 1.120) |
| ***Glioblastoma*** | | | |
| 31/12/2012  20 datapoints (1 in glioblastoma) | Treatment Effect Estimate | -0.128 (-0.213, -0.040) | 0.121 (-0.991, 1.217) |
|  | Within-Indication SD | 0.320 (0.043, 0.977) | 0.338 (0.016, 1.119) |
| 31/12/2013  26 datapoints (2 in glioblastoma) | Treatment Effect Estimate | -0.115 (-0.188, -0.042) | -0.010 (-0.615, 0.609) |
|  | Within-Indication SD | 0.192 (0.015, 0.729) | 0.257 (0.023, 0.931) |
| 31/12/2015  32 datapoints (3 in glioblastoma) | Treatment Effect Estimate | -0.114 (-0.174, -0.054) | -0.028 (-0.365, 0.323) |
|  | Within-Indication SD | 0.139 (0.009, 0.544) | 0.160 (0.011, 0.682) |

All reported estimates are the median and the corresponding 95% credible interval
**Abbreviations:** CPMA, common parameter meta-analysis; HR, hazard ratio; IPMA, independent parameter meta-analysis; NSCLC, non-small cell lung cancer; OFTPP, ovarian, fallopian tube and primary peritoneal cancer; RCC, renal cell carcinoma; SD, standard deviation.

**Table S5**. Synthesis results for progression-free survival. *Note: The treatment effect estimate is reported as the HR and corresponding 95% credible interval on the log-scale.*

| **Time Point** |  | **CPMA Model** | **IPMA Model** |
| --- | --- | --- | --- |
| ***Colorectal Cancer*** |  |  |  |
| 31/12/2003  5 datapoints (3 in colorectal cancer) | Treatment Effect Estimate | -0.653 (-0.975, -0.254) | -0.684 (-1.082, -0.289) |
|  | Within-Indication SD | 0.142 (0.006, 0.666) | 0.145 (0.006, 0.706) |
| 31/12/2004  6 datapoints (4 in colorectal cancer) | Treatment Effect Estimate | -0.592 (-0.850, -0.324) | -0.604 (-0.907, -0.343) |
|  | Within-Indication SD | 0.116 (0.005, 0.530) | 0.121 (0.005, 0.550) |
| 31/12/2006  9 datapoints (5 in colorectal cancer) | Treatment Effect Estimate | -0.463 (-0.662, -0.278) | -0.488 (-0.845, -0.200) |
|  | Within-Indication SD | 0.224 (0.070, 0.574) | 0.245 (0.075, 0.641) |
| 31/12/2009  20 datapoints (7 in colorectal cancer) | Treatment Effect Estimate | -0.357 (-0.489, -0.227) | -0.415 (-0.691, -0.190) |
|  | Within-Indication SD | 0.225 (0.096, 0.501) | 0.246 (0.109, 0.554) |
| 31/12/2010  21 datapoints (8 in colorectal cancer) | Treatment Effect Estimate | -0.380 (-0.512, -0.244) | -0.467 (-0.730, -0.245) |
|  | Within-Indication SD | 0.249 (0.120, 0.516) | 0.264 (0.132, 0.544) |
| 31/12/2011  26 datapoints (9 in colorectal cancer) | Treatment Effect Estimate | -0.393 (-0.510, -0.273) | -0.450 (-0.676, -0.265) |
|  | Within-Indication SD | 0.221 (0.109, 0.448) | 0.237 (0.118, 0.479) |
| 31/12/2012  29 datapoints (10 in colorectal cancer) | Treatment Effect Estimate | -0.393 (-0.497, -0.290) | -0.469 (-0.671, -0.297) |
|  | Within-Indication SD | 0.218 (0.112, 0.427) | 0.230 (0.122, 0.443) |
| ***Renal cell carcinoma*** | | | |
| 31/12/2003  5 datapoints (1 in RCC) | Treatment Effect Estimate | -0.653 (-0.973, -0.256) | -0.935 (-2.123, 0.261) |
|  | Within-Indication SD | 0.287 (0.013, 0.997) | 0.337 (0.016, 1.119) |
| 31/12/2008  14 datapoints (3 in RCC) | Treatment Effect Estimate | -0.408 (-0.546, -0.282) | -0.503 (-1.001, -0.152) |
|  | Within-Indication SD | 0.133 (0.005, 0.641) | 0.186 (0.008, 0.778) |
| 31/12/2009  20 datapoints (3 in RCC) | Treatment Effect Estimate | -0.357 (-0.490, -0.227) | -0.504 (-1.012, -0.147) |
|  | Within-Indication SD | 0.179 (0.008, 0.712) | 0.188 (0.008, 0.789) |
| ***Breast Cancer*** | | | |
| 31/12/2002  2 datapoints (1 in breast cancer) | Treatment Effect Estimate | -0.300 (-1.135, 0.317) | -0.018 (-1.130, 1.086) |
|  | Within-Indication SD | 0.387 (0.020, 1.110) | 0.338 (0.015, 1.123) |
| 31/12/2007  12 datapoints (2 in breast cancer) | Treatment Effect Estimate | -0.423 (-0.582, -0.271) | -0.249 (-1.014, 0.479) |
|  | Within-Indication SD | 0.331 (0.074, 0.902) | 0.366 (0.052, 1.032) |
| 31/12/2008  14 datapoints (4 in breast cancer) | Treatment Effect Estimate | -0.408 (-0.548, -0.281) | -0.333 (-0.667, -0.005) |
|  | Within-Indication SD | 0.199 (0.023, 0.586) | 0.216 (0.026, 0.670) |
| 31/12/2009  20 datapoints (8 in breast cancer) | Treatment Effect Estimate | -0.357 (-0.491, -0.226) | -0.212 (-0.459, 0.045) |
|  | Within-Indication SD | 0.308 (0.144, 0.611) | 0.288 (0.132, 0.591) |
| 31/12/2011  26 datapoints (9 in breast cancer) | Treatment Effect Estimate | -0.393 (-0.511, -0.273) | -0.212 (-0.421, 0.008) |
|  | Within-Indication SD | 0.310 (0.149, 0.591) | 0.257 (0.113, 0.521) |
| 31/12/2013  33 datapoints (11 in breast cancer) | Treatment Effect Estimate | -0.387 (-0.485, -0.285) | -0.220 (-0.377, -0.053) |
|  | Within-Indication SD | 0.272 (0.128, 0.506) | 0.210 (0.075, 0.418) |
| 31/12/2014  37 datapoints (12 in breast cancer) | Treatment Effect Estimate | -0.390 (-0.479, -0.299) | -0.233 (-0.377, -0.081) |
|  | Within-Indication SD | 0.257 (0.120, 0.469) | 0.198 (0.068, 0.390) |
| 31/12/2015  39 datapoints (13 in breast cancer) | Treatment Effect Estimate | -0.402 (-0.490, -0.309) | -0.236 (-0.373, -0.094) |
|  | Within-Indication SD | 0.258 (0.125, 0.462) | 0.189 (0.061, 0.367) |
| ***Non-small cell lung cancer*** | | | |
| 31/12/2005  8 datapoints (1 in NSCLC) | Treatment Effect Estimate | -0.551 (-0.774, -0.355) | -0.415 (-1.513, 0.686) |
|  | Within-Indication SD | 0.238 (0.012, 0.922) | 0.339 (0.016, 1.124) |
| 31/12/2007  13 datapoints (2 in NSCLC) | Treatment Effect Estimate | -0.423 (-0.572, -0.284) | -0.291 (-0.913, 0.329) |
|  | Within-Indication SD | 0.241 (0.037, 0.800) | 0.265 (0.030, 0.936) |
| 31/12/2013  35 datapoints (4 in NSCLC) | Treatment Effect Estimate | -0.392 (-0.485, -0.298) | -0.496 (-0.969, -0.067) |
|  | Within-Indication SD | 0.298 (0.108, 0.728) | 0.341 (0.134, 0.835) |
| 31/12/2014  37 datapoints (4 in NSCLC) | Treatment Effect Estimate | -0.390 (-0.479, -0.299) | -0.504 (-0.976, -0.073) |
|  | Within-Indication SD | 0.304 (0.113, 0.733) | 0.343 (0.136, 0.835) |
| 31/12/2018  43 datapoints (6 in NSCLC) | Treatment Effect Estimate | -0.399 (-0.483, -0.310) | -0.451 (-0.768, -0.152) |
|  | Within-Indication SD | 0.255 (0.096, 0.580) | 0.284 (0.113, 0.653) |
| 31/12/2019  43 datapoints (6 in NSCLC) | Treatment Effect Estimate | -0.394 (-0.478, -0.305) | -0.413 (-0.732, -0.107) |
|  | Within-Indication SD | 0.256 (0.097, 0.582) | 0.290 (0.113, 0.670) |
| ***Ovarian, fallopian tube and primary peritoneal cancer*** | | | |
| 31/12/2011  26 datapoints (3 in OFTPP) | Treatment Effect Estimate | -0.393 (-0.510, -0.273) | -0.558 (-1.091, -0.047) |
|  | Within-Indication SD | 0.313 (0.119, 0.795) | 0.330 (0.125, 0.887) |
| 31/12/2013  33 datapoints (4 in OFTPP) | Treatment Effect Estimate | -0.387 (-0.486, -0.286) | -0.433 (-0.908, 0.020) |
|  | Within-Indication SD | 0.323 (0.158, 0.739) | 0.369 (0.174, 0.859) |
| 31/12/2014  37 datapoints (5 in OFTPP) | Treatment Effect Estimate | -0.390 (-0.479, -0.300) | -0.437 (-0.800, -0.090) |
|  | Within-Indication SD | 0.283 (0.144, 0.630) | 0.318 (0.157, 0.728) |
| 31/12/2018  42 datapoints (7 in OFTPP) | Treatment Effect Estimate | -0.397 (-0.483, -0.305) | -0.415 (-0.712, -0.113) |
|  | Within-Indication SD | 0.272 (0.141, 0.573) | 0.300 (0.152, 0.646) |
| ***Cervical cancer*** | | | |
| 31/12/2014  37 datapoints (1 in cervical cancer) | Treatment Effect Estimate | -0.389 (-0.480, -0.300) | -0.385 (-1.485, 0.702) |
|  | Within-Indication SD | 0.161 (0.007, 0.832) | 0.337 (0.015, 1.114) |
| ***Glioblastoma*** | | | |
| 31/12/2012  29 datapoints (2 in glioblastoma) | Treatment Effect Estimate | -0.394 (-0.497, -0.291) | -0.348 (-0.926, 0.248) |
|  | Within-Indication SD | 0.150 (0.009, 0.670) | 0.231 (0.015, 0.910 |
| 31/12/2015  39 datapoints (3 in glioblastoma) | Treatment Effect Estimate | -0.403 (-0.490, -0.309) | -0.455 (-0.930, 0.011) |
|  | Within-Indication SD | 0.217 (0.040, 0.665) | 0.277 (0.065, 0.828) |

All reported estimates are the median and corresponding 95% credible interval
**Abbreviations:** CPMA, common parameter meta-analysis; HR, hazard ratio; IPMA, independent parameter meta-analysis; NSCLC; non-small cell lung cancer; OFTPP, ovarian, fallopian tube and primary peritoneal cancer; RCC, renal cell carcinoma; SD, standard deviation

## B-III: Model Fit Statistics

Model fit was compared across models using the DIC. Model fit statistics for the analyses with results presented in Tables S4 and S5 are reported in Table S6 and Table S7, respectively.

The DICs were similar across the two models, suggesting that both models were comparable in terms of fit to the data (differences lower than 3 points are not considered important).

**Table S6**. Model fit statistics for the overall survival analyses

| **Time Point** | **Statistic** | **CPMA Model** | **IPMA Model** |
| --- | --- | --- | --- |
| ***Colorectal cancer*** | | | |
| 31/12/2003  (2 datapoints) | DIC | 0.7704 | 0.7704 |
|  | pD | 1.647 | 1.647 |
|  | Deviance | -0.877 | -0.877 |
| 31/12/2004  (3 datapoints) | DIC | -1.354 | -1.354 |
|  | pD | 2.016 | 2.016 |
|  | Deviance | -3.370 | -3.370 |
| 31/12/2007  (7 datapoints) | DIC | -2.595 | -2.188 |
|  | pD | 5.562 | 6.053 |
|  | Deviance | -8.157 | -8.241 |
| 31/12/2009  (16 datapoints) | DIC | -4.478 | -3.861 |
|  | pD | 11.08 | 12.58 |
|  | Deviance | -15.558 | -16.441 |
| 31/12/2010  (17 datapoints) | DIC | -3.838 | -3.611 |
|  | pD | 11.69 | 13.06 |
|  | Deviance | -15.526 | -16.672 |
| 31/12/2011  (18 datapoints) | DIC | -5.385 | -5.289 |
|  | pD | 12.08 | 13.43 |
|  | Deviance | -17.461 | -18.722 |
| 31/12/2012  (20 datapoints) | DIC | -5.239 | -5.61 |
|  | pD | 13.32 | 14.63 |
|  | Deviance | -18.561 | -20.243 |
| ***Renal cell carcinoma*** | | | |
| 31/12/2008  (10 datapoints) | DIC | -4.182 | -3.248 |
|  | pD | 7.066 | 8.067 |
|  | Deviance | -11.248 | -11.315 |
| 31/12/2009  (16 datapoints) | DIC | -4.495 | -3.845 |
|  | pD | 11.09 | 12.58 |
|  | Deviance | -15.586 | -16.429 |
| ***Breast cancer*** | | | |
| 31/12/2007  (7 datapoints) | DIC | -2.577 | -2.161 |
|  | pD | 5.57 | 6.06 |
|  | Deviance | -8.147 | -8.224 |
| 31/12/2008  (11 datapoints) | DIC | -4.141 | -3.196 |
|  | pD | 7.079 | 8.098 |
|  | Deviance | -11.220 | -11.294 |
| 31/12/2009  (16 datapoints) | DIC | -4.479 | -3.899 |
|  | pD | 11.11 | 12.59 |
|  | Deviance | -15.585 | -16.494 |
| 31/12/2012  (20 datapoints) | DIC | -5.204 | -5.563 |
|  | pD | 13.32 | 14.68 |
|  | Deviance | -18.525 | -20.239 |
| 31/12/2013  (26 datapoints) | DIC | -10.18 | -9.537 |
|  | pD | 16.1 | 18.47 |
|  | Deviance | -26.284 | -28.003 |
| 31/12/2015  (32 datapoints) | DIC | -15.69 | -14.04 |
|  | pD | 17.12 | 20.11 |
|  | Deviance | -32.817 | -34.151 |
| ***Non-small cell lung cancer*** | | | |
| 31/12/2005  (4 datapoints) | DIC | -1.721 | -1.154 |
|  | pD | 2.692 | 3.021 |
|  | Deviance | -4.413 | -4.174 |
| 31/12/2007  (7 datapoints) | DIC | -2.596 | -2.193 |
|  | pD | 5.565 | 6.046 |
|  | Deviance | -8.161 | -8.239 |
| 31/12/2013  (26 datapoints) | DIC | -10.18 | -9.502 |
|  | pD | 16.06 | 18.49 |
|  | Deviance | -26.242 | -27.994 |
| 31/12/2017  (33 datapoints) | DIC | -15.97 | -14.11 |
|  | pD | 17.35 | 20.58 |
|  | Deviance | -33.319 | -34.686 |
| 31/12/2018  (37 datapoints) | DIC | -16.68 | -14.87 |
|  | pD | 17.72 | 20.92 |
|  | Deviance | -34.395 | -35.785 |
| 31/12/2019  (38 datapoints) | DIC | -17.27 | -15.28 |
|  | pD | 17.85 | 21.13 |
|  | Deviance | -35.120 | -36.413 |
| ***Ovarian, fallopian tube and primary peritoneal cancer*** | | | |
| 31/12/2013  (26 datapoints) | DIC | -10.21 | -9.538 |
|  | pD | 16.11 | 18.43 |
|  | Deviance | -26.316 | -27.963 |
| 31/12/2014  (29 datapoints) | DIC | -12.65 | -10.87 |
|  | pD | 16.91 | 19.98 |
|  | Deviance | -29.563 | -30.843 |
| 31/12/2018  (37 datapoints) | DIC | -16.62 | -14.81 |
|  | pD | 17.72 | 20.91 |
|  | Deviance | -34.340 | -35.712 |
| ***Cervical cancer*** | | | |
| 31/12/2014  (29 datapoints) | DIC | -12.61 | -10.86 |
|  | pD | 17.02 | 19.97 |
|  | Deviance | -29.628 | -30.831 |
| ***Glioblastoma*** | | | |
| 31/12/2012  (20 datapoints) | DIC | -5.28 | -5.558 |
|  | pD | 13.30 | 14.69 |
|  | Deviance | -18.579 | -20.244 |
| 31/12/2013  (26 datapoints) | DIC | -10.200 | -9.432 |
|  | pD | 16.13 | 18.46 |
|  | Deviance | -26.336 | -27.894 |
| 31/12/2015  (32 datapoints) | DIC | -15.68 | -13.95 |
|  | pD | 17.07 | 20.23 |
|  | Deviance | -32.745 | -34.178 |

**Abbreviations:** CPMA, common parameter meta-analysis; IP, independent parameter meta-analysis; NSCLC; non-small cell lung cancer.

**Table S7**. Model fit statistics for the progression-free survival analyses

| **Time Point** | **Statistic** | **CPMA Model** | **IPMA Model** |
| --- | --- | --- | --- |
| ***Colorectal cancer*** | | | |
| 31/12/2003  (5 datapoints) | DIC | 4.659 | 5.038 |
|  | pD | 3.342 | 3.734 |
|  | Deviance | 1.317 | 1.305 |
| 31/12/2004  (6 datapoints) | DIC | 3.717 | 3.894 |
|  | pD | 3.758 | 4.118 |
|  | Deviance | -0.042 | -0.224 |
| 31/12/2006  (9 datapoints) | DIC | 3.273 | 3.552 |
|  | pD | 7.066 | 7.758 |
|  | Deviance | -3.793 | -4.207 |
| 31/12/2009  (20 datapoints) | DIC | -1.918 | -2.69 |
|  | pD | 16.67 | 17.28 |
|  | Deviance | -18.588 | -19.969 |
| 31/12/2010  (21 datapoints) | DIC | -1.906 | -2.74 |
|  | pD | 17.47 | 18.04 |
|  | Deviance | -19.38 | -20.782 |
| 31/12/2011  (26 datapoints) | DIC | -5.401 | -5.998 |
|  | pD | 21.68 | 22.34 |
|  | Deviance | -27.085 | -28.333 |
| 31/12/2012  (29 datapoints) | DIC | -7.013 | -7.575 |
|  | pD | 24.00 | 24.98 |
|  | Deviance | -31.015 | -32.555 |
| ***Renal cell carcinoma*** | | | |
| 31/12/2003  (5 datapoints) | DIC | 4.672 | 5.078 |
|  | pD | 3.349 | 3.756 |
|  | Deviance | 1.323 | 1.322 |
| 31/12/2008  (14 datapoints) | DIC | 0.2667 | 0.4393 |
|  | pD | 10.91 | 12.06 |
|  | Deviance | -10.646 | -11.621 |
| 31/12/2009  (20 datapoints) | DIC | -1.88 | -2.709 |
|  | pD | 16.7 | 17.25 |
|  | Deviance | -18.577 | -19.964 |
| ***Breast cancer*** | | | |
| 31/12/2002  (2 datapoints) | DIC | 3.903 | 3.682 |
|  | pD | 1.948 | 2.002 |
|  | Deviance | 1.956 | 1.679 |
| 31/12/2007  (12 datapoints) | DIC | 1.571 | 1.968 |
|  | pD | 9.542 | 10.55 |
|  | Deviance | -7.972 | -8.579 |
| 31/12/2008  (14 datapoints) | DIC | 0.3024 | 0.3527 |
|  | pD | 10.94 | 12.03 |
|  | Deviance | -10.634 | -11.678 |
| 31/12/2009  (20 datapoints) | DIC | -1.929 | -2.704 |
|  | pD | 16.69 | 17.28 |
|  | Deviance | -18.619 | -19.985 |
| 31/12/2011  (26 datapoints) | DIC | -5.35 | -5.935 |
|  | pD | 21.71 | 22.34 |
|  | Deviance | -27.061 | -28.275 |
| 31/12/2013  (33 datapoints) | DIC | -9.362 | -10.05 |
|  | pD | 27.45 | 28.03 |
|  | Deviance | -36.811 | -38.086 |
| 31/12/2014  (37 datapoints) | DIC | -10.65 | -10.70 |
|  | pD | 30.17 | 31.11 |
|  | Deviance | -40.825 | -41.816 |
| 31/12/2015  (39 datapoints) | DIC | -10.66 | -10.74 |
|  | pD | 31.72 | 32.32 |
|  | Deviance | -42.376 | -43.065 |
| ***Non-small cell lung cancer*** | | | |
| 31/12/2005  (8 datapoints) | DIC | 4.078 | 4.92 |
|  | pD | 5.435 | 6.222 |
|  | Deviance | -1.357 | -1.302 |
| 31/12/2007  (13 datapoints) | DIC | 1.09 | 1.498 |
|  | pD | 10.29 | 11.39 |
|  | Deviance | -9.196 | -9.887 |
| 31/12/2013  (35 datapoints) | DIC | -9.238 | -9.029 |
|  | pD | 28.71 | 29.8 |
|  | Deviance | -37.952 | -38.83 |
| 31/12/2014  (37 datapoints) | DIC | -10.69 | -10.54 |
|  | pD | 30.16 | 31.16 |
|  | Deviance | -40.848 | -41.693 |
| 31/12/2018  (43 datapoints) | DIC | -7.556 | -6.862 |
|  | pD | 33.33 | 34.37 |
|  | Deviance | -40.889 | -41.231 |
| 31/12/2019  (43 datapoints) | DIC | -7.415 | -7.059 |
|  | pD | 33.44 | 34.45 |
|  | Deviance | -40.855 | -41.510 |
| ***Ovarian, fallopian tube and primary peritoneal cancer*** | | | |
| 31/12/2011  (26 datapoints) | DIC | -5.382 | -6.008 |
|  | pD | 21.68 | 22.33 |
|  | Deviance | -27.066 | -28.336 |
| 31/12/2013  (33 datapoints) | DIC | -9.312 | -10.12 |
|  | pD | 27.47 | 28.04 |
|  | Deviance | -36.778 | -38.161 |
| 31/12/2014  (37 datapoints) | DIC | -10.75 | -10.56 |
|  | pD | 30.15 | 31.13 |
|  | Deviance | -40.898 | -41.692 |
| 31/12/2018  (42 datapoints) | DIC | -7.346 | -7.043 |
|  | pD | 32.87 | 33.75 |
|  | Deviance | -40.216 | -40.796 |
| ***Cervical cancer*** | | | |
| 31/12/2014  (37 datapoints) | DIC | -10.70 | -10.64 |
|  | pD | 30.16 | 31.14 |
|  | Deviance | -40.853 | -41.784 |
| ***Glioblastoma*** | | | |
| 31/12/2012  (29 datapoints) | DIC | -6.988 | -7.521 |
|  | pD | 24.01 | 24.98 |
|  | Deviance | -30.999 | -32.500 |
| 31/12/2015  (39 datapoints) | DIC | -10.75 | -10.70 |
|  | pD | 31.70 | 32.33 |
|  | Deviance | -42.447 | -43.027 |

**Abbreviations:** CPMA, common parameter meta-analysis; IPMA, independent parameter meta-analysis; NSCLC; non-small cell lung cancer.

## B-IV: Results of the Synthesis Models- Target Indication

**Table S8**. Synthesis results for overall survival for breast cancer and ovarian cancer using the common parameter and independent parameter meta-analysis models. *Note: The treatment effect estimate is reported as the HR and corresponding 95% credible interval on the log-scale.*

|  |  | **CPMA Model** | **IPMA Model** |
| --- | --- | --- | --- |
| ***Breast cancer*** | | | |
| Time Point 1  (31/12/2009) | Treatment Effect Estimate | -0.114 (-0.209, -0.016) | -0.019 (-0.211, 0.239) |
|  | Within-Indication SD | 0.133 (0.006, 0.487) | 0.152 (0.007, 0.520) |
| Time Point 2  (31/12/2012) | Treatment Effect Estimate | -0.127 (-0.201, -0.052) | -0.019 (-0.209, 0.236) |
|  | Within-Indication SD | 0.139 (0.006, 0.493) | 0.150 (0.008, 0.511) |
| Time Point 3  (31/12/2021) | Treatment Effect Estimate | -0.104 (-0.159, -0.049) | -0.054 (-0.167, 0.080) |
|  | Within-Indication SD | 0.063 (0.002, 0.258) | 0.076 (0.002, 0.285) |
| ***Ovarian, fallopian tube and primary peritoneal cancer*** | | | |
| Time Point 1  (31/12/2011) | Treatment Effect Estimate | -0.124 (-0.200, -0.046) | -0.100 (-0.379, 0.205) |
|  | Within-Indication SD | 0.075 (0.003, 0.418) | 0.109 (0.004, 0.611) |
| Time Point 2  (31/12/2015) | Treatment Effect Estimate | -0.114 (-0.175, -0.053) | -0.090 (-0.285, 0.090) |
|  | Within-Indication SD | 0.070 (0.003, 0.310) | 0.086 (0.004, 0.423) |
| Time Point 3  (31/12/2021) | Treatment Effect Estimate | -0.105 (-0.160, -0.048) | -0.071 (-0.191, 0.041) |
|  | Within-Indication SD | 0.055 (0.003, 0.221) | 0.056 (0.003, 0.258) |

**Abbreviations:** CPMA, common parameter meta-analysis; IPMA, independent parameter meta-analysis; SD, standard deviation

**Table S9**. Synthesis results for progression-free survival for breast cancer and ovarian cancer using the common parameter and independent parameter meta-analysis models. *Note: The treatment effect estimate is reported as the HR and corresponding 95% credible interval on the log-scale.*

|  |  | **CPMA Model** | **IPMA Model** |
| --- | --- | --- | --- |
| ***Breast cancer*** | | | |
| Time Point 1  (31/12/2009) | Treatment Effect Estimate | -0.357 (-0.489, -0.227) | -0.212 (-0.457, 0.046) |
|  | Within-Indication SD | 0.307 (0.145, 0.608) | 0.288 (0.132, 0.588) |
| Time Point 2  (31/12/2012) | Treatment Effect Estimate | -0.386 (-0.481, -0.292) | -0.213 (-0.422, 0.006) |
|  | Within-Indication SD | 0.304 (0.150, 0.576) | 0.257 (0.111, 0.517) |
| Time Point 3  (31/12/2021) | Treatment Effect Estimate | -0.394 (-0.480, -0.305) | -0.236 (-0.372, -0.095) |
|  | Within-Indication SD | 0.252 (0.122, 0.451) | 0.189 (0.056, 0.365) |
| ***Ovarian, fallopian tube and primary peritoneal cancer*** | | | |
| Time Point 1  (31/12/2011) | Treatment Effect Estimate | -0.382 (-0.495, -0.267) | -0.449 (-0.903, -0.013) |
|  | Within-Indication SD | 0.304 (0.142, 0.709) | 0.347 (0.157, 0.833) |
| Time Point 2  (31/12/2015) | Treatment Effect Estimate | -0.402 (-0.49, -0.309) | -0.436 (-0.800, -0.092) |
|  | Within-Indication SD | 0.282 (0.143, 0.623) | 0.318 (0.156, 0.726) |
| Time Point 3  (31/12/2021) | Treatment Effect Estimate | -0.394 (-0.478, -0.305) | -0.415 (-0.713, -0.113) |
|  | Within-Indication SD | 0.273 (0.141, 0.575) | 0.299 (0.152, 0.644) |

**Abbreviations:** CPMA, common parameter meta-analysis; IPMA, independent parameter meta-analysis; SD, standard deviation

**Table S10**. Model fit statistics for the overall survival analyses for breast and ovarian cancer using the common parameter and independent parameter meta-analysis model

|  |  | **CPMA Model** | **IPMA Model** |
| --- | --- | --- | --- |
| ***Breast cancer*** | | | |
| Time Point 1  (31/12/2009)  20 datapoints | DIC | -1.886 | -2.772 |
|  | pD | 16.69 | 17.26 |
|  | Deviance | -18.577 | -19.983 |
| Time Point 2  (31/12/2012)  31 datapoints | DIC | -8.574 | -8.444 |
|  | pD | 25.54 | 26.95 |
|  | Deviance | -34.119 | -35.398 |
| Time Point 3  (31/12/2021)  43 datapoints | DIC | -7.510 | -6.976 |
|  | pD | 33.43 | 34.48 |
|  | Deviance | -40.935 | -41.455 |
| ***Ovarian, fallopian tube and primary peritoneal cancer*** | | | |
| Time Point 1  (31/12/2011)  27 datapoints | DIC | -6.805 | -7.643 |
|  | pD | 22.65 | 23.27 |
|  | Deviance | -29.454 | -30.918 |
| Time Point 2  (31/12/2015)  39 datapoints | DIC | -10.580 | -10.670 |
|  | pD | 31.74 | 32.35 |
|  | Deviance | -42.318 | -43.027 |
| Time Point 3  (31/12/2021)  43 datapoints | DIC | -7.462 | -7.134 |
|  | pD | 33.44 | 34.45 |
|  | Deviance | -40.906 | -45.583 |

**Table S11**. Model fit statistics for the overall survival analyses for breast and ovarian cancer using the common parameter and independent parameter meta-analysis model

|  |  | **CPMA Model** | **IPMA Model** |
| --- | --- | --- | --- |
| ***Breast cancer*** | | | |
| Time Point 1  (31/12/2009)  16 datapoints | DIC | -4.513 | -3.886 |
|  | pD | 11.10 | 12.59 |
|  | Deviance | -15.615 | -16.478 |
| Time Point 2  (31/12/2012)  24 datapoints | DIC | -7.178 | -6.319 |
|  | pD | 15.37 | 17.74 |
|  | Deviance | -22.544 | -24.058 |
| Time Point 3  (31/12/2021)  38 datapoints | DIC | -17.300 | -15.200 |
|  | pD | 17.88 | 21.18 |
|  | Deviance | -35.188 | -36.385 |
| ***Ovarian, fallopian tube and primary peritoneal cancer*** | | | |
| Time Point 1  (31/12/2011)  21 datapoints | DIC | -8.533 | -7.256 |
|  | pD | 13.32 | 15.54 |
|  | Deviance | -21.853 | -22.792 |
| Time Point 2  (31/12/2015)  32 datapoints | DIC | -15.610 | -13.930 |
|  | pD | 17.08 | 20.18 |
|  | Deviance | -32.689 | -34.106 |
| Time Point 3  (31/12/2021)  38 datapoints | DIC | -17.290 | -15.080 |
|  | pD | 17.89 | 21.2 |
|  | Deviance | -35.180 | -36.277 |

# C: Key Features of Oncology Evidence

## C-I: Maturity

Maturity for OS and PFS are shown in Tables S12 and S13, respectively. The tables only include studies for which maturity could be calculated.

**Table S12.** Maturity for OS

| **Trial** | **Timepoint** | **Treatment Arm** | **Total Trial Participants** | **Number of Events** | **Maturity Ratio** |
| --- | --- | --- | --- | --- | --- |
| ***Colorectal Cancer*** | | | | | |
| HORIZON III | 15/11/2009 | Chemotherapy | 709 | 239 | 0.337 |
|  |  | Bevacizumab | 713 | 247 | 0.346 |
| ***Renal cell carcinoma*** | | | | | |
| AVOREN | 01/08/2006 | Chemotherapy | 322 | 137 | 0.425 |
|  |  | Bevacizumab | 327 | 114 | 0.349 |
|  | 01/09/2008 | Chemotherapy | 322 | 224 | 0.696 |
|  |  | Bevacizumab | 327 | 220 | 0.673 |
| ***Breast cancer*** | | | | | |
| RIBBON-2 | 01/03/2009 | Chemotherapy | 255 | 109 | 0.427 |
|  |  | Bevacizumab | 459 | 206 | 0.449 |
| AVADO | 01/04/2009 | Chemotherapy | 241 | 133 | 0.552 |
|  |  | Bevacizumab | 247 | 131 | 0.530 |
| SUN1094 | 01/06/2009 | Chemotherapy | 242 | 52 | 0.215 |
|  |  | Bevacizumab | 243 | 32 | 0.132 |
| LEA | 01/12/2013 | Chemotherapy | 184 | 46 | 0.250 |
|  |  | Bevacizumab | 190 | 47 | 0.247 |
| TANIA | 30/04/2015 | Chemotherapy | 247 | 156 | 0.632 |
|  |  | Bevacizumab | 247 | 163 | 0.660 |
| MERiDiAN | 30/11/2014 | Chemotherapy | 233 | 105 | 0.451 |
|  |  | Bevacizumab | 238 | 91 | 0.382 |
| ***Non-small cell lung cancer*** | | | | | |
| E4599 | 01/10/2005 | Chemotherapy | 444 | 344 | 0.775 |
|  |  | Bevacizumab | 434 | 305 | 0.703 |
| AVAiL | 01/11/2007 | Chemotherapy | 347 | 240 | 0.692 |
|  |  | Bevacizumab | 351 | 242 | 0.689 |
| ***Ovarian, fallopian tube, and primary peritoneal cancer*** | | | | | |
| GOG218 | 01/02/2010 | Chemotherapy | 625 | 156 | 0.250 |
|  |  | Bevacizumab | 623 | 138 | 0.222 |
|  | 01/08/2011 | Chemotherapy | 625 | 298 | 0.477 |
|  |  | Bevacizumab | 623 | 269 | 0.432 |
| ICON7 | 01/02/2010 | Chemotherapy | 764 | 130 | 0.170 |
|  |  | Bevacizumab | 764 | 111 | 0.145 |
|  | 01/11/2010 | Chemotherapy | 764 | 200 | 0.262 |
|  |  | Bevacizumab | 764 | 178 | 0.233 |
| GOG213 | 01/11/2014 | Chemotherapy | 337 | 214 | 0.635 |
|  |  | Bevacizumab | 337 | 201 | 0.596 |
| AURELIA | 01/01/2013 | Chemotherapy | 182 | 136 | 0.747 |
|  |  | Bevacizumab | 179 | 128 | 0.715 |
| ***Cervical cancer*** | | | | | |
| GOG240 | 01/03/2012 | Chemotherapy | 225 | 140 | 0.622 |
|  |  | Bevacizumab | 227 | 131 | 0.577 |
|  | 01/03/2014 | Chemotherapy | 225 | 175 | 0.778 |
|  |  | Bevacizumab | 227 | 173 | 0.762 |
| ***Glioblastoma*** | | | | | |
| RTOG0825 | 01/12/2012 | Chemotherapy | 317 | 320 | 1.009 |
|  |  | Bevacizumab | 198 | 215 | 1.086 |
| EORTC26101 | 01/10/2015 | Chemotherapy | 149 | 113 | 0.758 |
|  |  | Bevacizumab | 288 | 216 | 0.750 |

**Table S13.** Maturity for PFS

| **Trial** | **Timepoint** | **Treatment Arm** | **Total Trial Participants** | **Number of Events** | **Maturity Ratio** |
| --- | --- | --- | --- | --- | --- |
| ***Colorectal Cancer*** | | | | | |
| AVF0780 | 01/10/2000 | Chemotherapy | 36 | 26 | 0.722 |
|  |  | Bevacizumab | 35 | 22 | 0.629 |
| HORIZON III | 15/11/2009 | Chemotherapy | 709 | 471 | 0.664 |
|  |  | Bevacizumab | 713 | 453 | 0.635 |
| ***Renal cell carcinoma*** | | | | | |
| AVOREN | 01/08/2006 | Chemotherapy | 322 | 275 | 0.854 |
|  |  | Bevacizumab | 327 | 230 | 0.703 |
| ***Breast cancer*** | | | | | |
| E2100 | 01/02/2005 | Chemotherapy | 354 | 244 | 0.689 |
|  |  | Bevacizumab | 368 | 201 | 0.546 |
|  | 01/04/2005 | Chemotherapy | 354 | 184 | 0.520 |
|  |  | Bevacizumab | 368 | 173 | 0.470 |
|  | 01/06/2007 | Chemotherapy | 326 | 308 | 0.945 |
|  |  | Bevacizumab | 347 | 316 | 0.911 |
| RIBBON-2 | 01/03/2009 | Chemotherapy | 255 | 184 | 0.722 |
|  |  | Bevacizumab | 459 | 372 | 0.810 |
| AVADO | 01/04/2009 | Chemotherapy | 241 | 219 | 0.909 |
|  |  | Bevacizumab | 247 | 220 | 0.891 |
| AVEREL | 30/06/2011 | Chemotherapy | 208 | 154 | 0.740 |
|  |  | Bevacizumab | 216 | 153 | 0.708 |
| SUN1094 | 01/06/2009 | Chemotherapy | 242 | 89 | 0.368 |
|  |  | Bevacizumab | 243 | 70 | 0.288 |
| Martin (2011) | 01/05/2009 | Chemotherapy | 94 | 15 | 0.16 |
|  |  | Bevacizumab | 97 | 9 | 0.093 |
| LEA | 01/12/2013 | Chemotherapy | 184 | 135 | 0.734 |
|  |  | Bevacizumab | 190 | 128 | 0.674 |
| TANIA | 20/12/2013 | Chemotherapy | 247 | 203 | 0.822 |
|  |  | Bevacizumab | 247 | 204 | 0.826 |
| MERiDiAN | 30/11/2014 | Chemotherapy | 233 | 168 | 0.721 |
|  |  | Bevacizumab | 238 | 152 | 0.639 |
| ***Non-small cell lung cancer*** | | | | | |
| E4599 | 01/10/2005 | Chemotherapy | 444 | 405 | 0.912 |
|  |  | Bevacizumab | 434 | 374 | 0.862 |
| JO25567 | 01/06/2013 | Chemotherapy | 77 | 57 | 0.740 |
|  |  | Bevacizumab | 75 | 46 | 0.613 |
| ***Ovarian, fallopian tube, and primary peritoneal cancer*** | | | | | |
| ICON7 | 01/02/2010 | Chemotherapy | 764 | 392 | 0.513 |
|  |  | Bevacizumab | 764 | 367 | 0.480 |
|  | 01/11/2010 | Chemotherapy | 764 | 392 | 0.513 |
|  |  | Bevacizumab | 764 | 367 | 0.480 |
|  | 01/03/2013 | Chemotherapy | 764 | 526 | 0.688 |
|  |  | Bevacizumab | 764 | 554 | 0.725 |
| OCEANS | 01/08/2011 | Chemotherapy | 242 | 187 | 0.773 |
|  |  | Bevacizumab | 242 | 151 | 0.624 |
| AURELIA | 01/11/2011 | Chemotherapy | 182 | 166 | 0.912 |
|  |  | Bevacizumab | 179 | 135 | 0.754 |
| ***Cervical cancer*** | | | | | |
| GOG240 | 01/03/2012 | Chemotherapy | 225 | 184 | 0.818 |
|  |  | Bevacizumab | 227 | 183 | 0.806 |
|  | 01/03/2014 | Chemotherapy | 225 | 206 | 0.916 |
|  |  | Bevacizumab | 227 | 199 | 0.877 |
| ***Glioblastoma*** | | | | | |
| RTOG0825 | 01/12/2012 | Chemotherapy | 317 | 256 | 0.808 |
|  |  | Bevacizumab | 320 | 256 | 0.800 |
| AvaGlio | 01/03/2012 | Chemotherapy | 463 | 387 | 0.836 |
|  |  | Bevacizumab | 458 | 354 | 0.773 |
| EORTC26101 | 01/10/2015 | Chemotherapy | 149 | 143 | 0.960 |
|  |  | Bevacizumab | 288 | 260 | 0.903 |

## C-II: Precision

**Table S14.** Precision for PFS and OS, measured as SE/|ln(HR)|

| **Trial** | **Time Point** | **Outcome** | **HR** | **SE of ln(HR)** | **SE/|ln(HR)|** |
| --- | --- | --- | --- | --- | --- |
| ***Colorectal cancer*** | | | | | |
| AVF0780 | 01/10/2000 | PFS | 0.46 | 0.274 | 0.353 |
| AVF2192 | 01/09/2003 | PFS | 0.50 | 0.195 | 0.281 |
|  |  | OS | 0.79 | 0.172 | 0.731 |
| AVF2107 | 01/04/2003 | PFS | 0.54 | 0.190 | 0.309 |
|  |  | OS | 0.66 | 0.122 | 0.294 |
| E3200 | 01/05/2004 | PFS | 0.61 | 0.124 | 0.251 |
|  |  | OS | 0.75 | 0.088 | 0.306 |
| NO16966 | 01/02/2006 | PFS | 0.83 | 0.071 | 0.380 |
|  | 01/02/2007 | OS | 0.89 | 0.078 | 0.665 |
| MAX | 27/02/2009 | PFS | 0.62 | 0.122 | 0.255 |
|  |  | OS | 0.88 | 0.130 | 1.014 |
| ML18147 | 01/05/2011 | PFS | 0.67 | 0.076 | 0.189 |
|  |  | OS | 0.83 | 0.080 | 0.427 |
| HORIZON-III | 15/11/2009 | PFS | 0.91 | 0.064 | 0.684 |
|  |  | OS | 1.05 | 0.075 | 1.533 |
| AVEX | 19/01/2012 | PFS | 0.53 | 0.133 | 0.209 |
|  |  | OS | 0.79 | 0.165 | 0.702 |
| ARTIST | 01/12/2010 | PFS | 0.44 | 0.181 | 0.220 |
|  |  | OS | 0.62 | 0.214 | 0.448 |
| ***Renal cell carcinoma*** | | | | | |
| AVF0890 | 01/02/2003 | PFS | 0.39 | 0.284 | 0.303 |
| CALGB-90206 | 01/10/2007 | PFS | 0.67 | 0.083 | 0.208 |
|  | 01/03/2009 | OS | 0.86 | 0.105 | 0.694 |
| AVOREN | 01/08/2006 | PFS | 0.61 | 0.091 | 0.185 |
|  |  | OS | 0.75 | 0.131 | 0.456 |
|  | 01/09/2008 | OS | 0.86 | 0.082 | 0.541 |
| ***Breast cancer*** | | | | | |
| AVF2119 | 01/06/2002 | PFS | 0.98 | 0.124 | 6.118 |
| E2100 | 01/02/2005 | PFS | 0.42 | 0.104 | 0.120 |
|  | 01/04/2005 | PFS | 0.48 | 0.187 | 0.255 |
|  | 01/10/2006 | OS | 0.88 | 0.079 | 0.619 |
|  | 01/06/2007 | PFS | 0.60 | 0.155 | 0.303 |
|  |  | OS | 0.87 | 0.096 | 0.691 |
| RIBBON-1 | 01/07/2008 | PFS | 0.67 | 0.050 | 0.123 |
|  |  | OS | 0.92 | 0.100 | 1.200 |
| RIBBON-2 | 01/03/2009 | PFS | 0.78 | 0.098 | 0.395 |
|  |  | OS | 0.90 | 0.121 | 1.146 |
| AVADO | 01/10/2007 | PFS | 0.61 | 0.124 | 0.251 |
|  | 01/04/2009 | PFS | 0.77 | 0.095 | 0.365 |
|  |  | OS | 1.03 | 0.164 | 5.539 |
| AVEREL | 30/06/2011 | PFS | 0.82 | 0.115 | 0.579 |
| SUN1094 | 01/06/2009 | PFS | 1.63 | 0.165 | 0.337 |
|  |  | OS | 1.82 | 0.230 | 0.384 |
| Martin (2011) | 01/05/2009 | PFS | 0.79 | 0.202 | 0.857 |
| LEA | 01/12/2013 | PFS | 0.83 | 0.125 | 0.670 |
|  |  | OS | 0.87 | 0.210 | 1.506 |
| E1105 | 01/10/2015 | PFS | 0.73 | 0.268 | 0.852 |
|  |  | OS | 1.09 | 0.299 | 3.470 |
| TANIA | 20/12/2013 | PFS | 0.75 | 0.108 | 0.374 |
|  | 30/04/2015 | OS | 0.96 | 0.119 | 2.906 |
| MERiDiAN | 30/11/2014 | PFS | 0.68 | 0.148 | 0.383 |
|  |  | OS | 0.81 | 0.146 | 0.692 |
| ***Non-small cell lung cancer*** | | | | | |
| E4599 | 01/10/2005 | PFS | 0.66 | 0.077 | 0.185 |
|  |  | OS | 0.79 | 0.081 | 0.343 |
| AVAiL | 01/10/2006 | PFS | 0.82 | 0.093 | 0.470 |
|  | 01/11/2007 | PFS | 0.85 | 0.080 | 0.494 |
|  |  | OS | 1.03 | 0.091 | 3.088 |
| JO25567 | 01/06/2013 | PFS | 0.54 | 0.200 | 0.325 |
|  | 01/03/2014 | PFS | 0.52 | 0.198 | 0.302 |
|  | 01/10/2017 | OS | 0.81 | 0.215 | 1.019 |
| BEYOND | 27/01/2013 | PFS | 0.40 | 0.159 | 0.173 |
|  |  | OS | 0.68 | 0.158 | 0.410 |
| IMpower150 | 01/01/2018 | PFS | 0.88 | 0.228 | 1.785 |
|  |  | OS | 1.08 | 0.302 | 3.924 |
| NEJ026 | 01/09/2017 | PFS | 0.61 | 0.190 | 0.377 |
|  | 01/11/2019 | PFS | 0.77 | 0.163 | 0.633 |
|  |  | OS | 1.01 | 0.200 | 28.634 |
| ***Ovarian, fallopian tube, and primary peritoneal cancer*** | | | | | |
| GOG218 | 01/02/2010 | PFS | 0.72 | 0.071 | 0.212 |
|  |  | OS | 0.92 | 0.117 | 1.322 |
|  | 01/08/2011 | PFS | 0.77 | 0.062 | 4.183 |
|  |  | OS | 0.89 | 0.074 | 0.606 |
|  | 01/01/2018 | OS | 0.96 | 0.063 | 1.554 |
| ICON7 | 01/02/2010 | PFS | 0.81 | 0.075 | 0.357 |
|  |  | OS | 0.81 | 0.128 | 0.607 |
|  | 01/11/2010 | PFS | 0.87 | 0.064 | 2.172 |
|  |  | OS | 0.85 | 0.089 | 0.549 |
|  | 01/03/2013 | PFS | 0.93 | 0.060 | 0.826 |
|  |  | OS | 0.99 | 0.075 | 7.451 |
| OCEANS | 01/09/2010 | OS | 0.75 | 0.172 | 0.599 |
|  | 01/08/2011 | PFS | 0.48 | 0.113 | 0.156 |
|  |  | OS | 1.03 | 0.138 | 5.161 |
|  | 01/07/2013 | OS | 0.95 | 0.108 | 2.198 |
| GOG213 | 01/11/2014 | PFS | 0.63 | 0.083 | 0.178 |
|  |  | OS | 0.83 | 0.099 | 0.525 |
| AURELIA | 01/11/2011 | PFS | 0.48 | 0.117 | 0.159 |
|  | 01/01/2013 | OS | 0.85 | 0.126 | 0.773 |
| mEOC/GOG241 | 01/02/2018 | PFS | 0.64 | 0.280 | 0.631 |
|  |  | OS | 0.89 | 0.392 | 3.300 |
| ***Cervical cancer*** | | | | | |
| GOG240 | 01/03/2012 | PFS | 0.67 | 0.107 | 0.266 |
|  |  | OS | 0.71 | 0.144 | 0.421 |
|  | 01/03/2014 | PFS | 0.68 | 0.103 | 0.268 |
|  |  | OS | 0.77 | 0.084 | 0.322 |
| ***Glioblastoma*** | | | | | |
| RTOG0825 | 01/12/2012 | PFS | 0.79 | 0.090 | 0.383 |
|  |  | OS | 1.13 | 0.099 | 0.809 |
| AvaGlio | 01/03/2012 | PFS | 0.64 | 0.076 | 0.170 |
|  | 01/02/2013 | OS | 0.88 | 0.075 | 0.587 |
| EORTC26101 | 01/10/2015 | PFS | 0.49 | 0.114 | 0.160 |
|  |  | OS | 0.95 | 0.125 | 2.445 |

# D: Additional Figures

**Abbreviation table for all figures**

| **Abbreviation** | **Definition** |
| --- | --- |
| BEV | Bevacizumab |
| BRE | Breast cancer |
| CER | Cervical cancer |
| CHM | Chemotherapy |
| CI | Confidence interval |
| COL | Colorectal cancer |
| CP | Common parameter |
| Comp | Comparator |
| GLIO | Glioblastoma |
| HMA | Hierarchical meta-analysis |
| HOR | Hormonal therapy |
| HR | Hazard ratio |
| IMM | Immunotherapy |
| IP | Independent parameter |
| NSCLC | Non-small cell lung cancer |
| OFTPP^1^ | Ovarian, fallopian tube, and primary peritoneal cancer |
| OS | Overall survival |
| PBO | Placebo |
| PFS | Progression-free survival |
| RAD | Radiotherapy |
| REN | Renal cell carcinoma |
| SE | Standard error |
| TAR | Targeted therapy |

^1^ These three cancers were also collectively referred to as ‘ovarian cancer’.


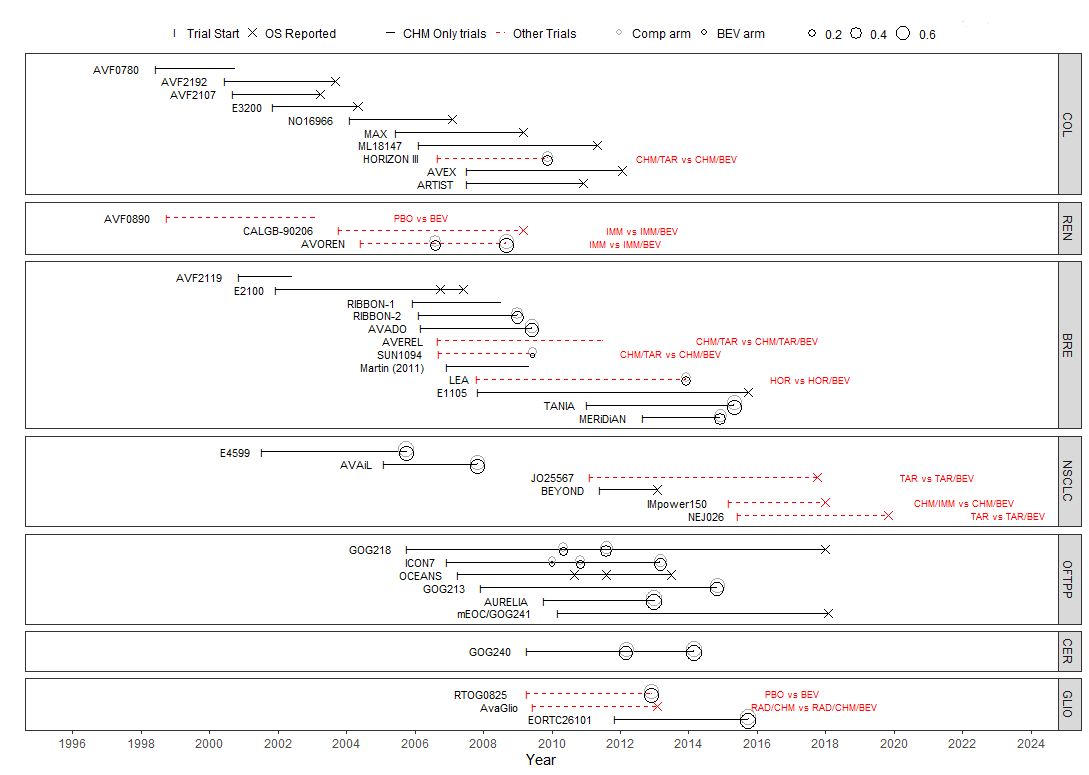
**Figure S2**. Modified timeline plot showing the maturity of OS evidence

An arbitrary gap of 3-4 months was added to improve visibility, where necessary. A gap 4 months was introduced between AVADO and RIBBON-2 by moving the marker for the final AVADO datapoint 2 months later, and 2 months earlier for RIBBON-2.

**Figure S3**. Modified timeline plot showing the maturity of PFS evidence


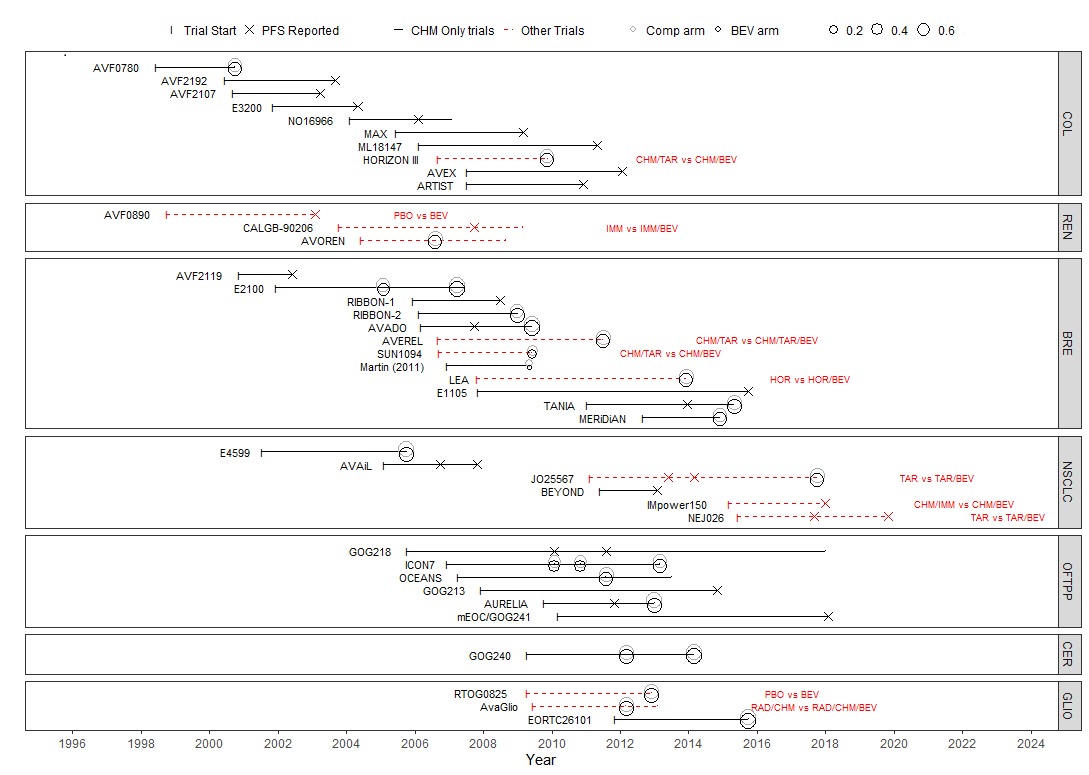


An arbitrary gap of 3-4 months was added to improve visibility, where necessary. A gap 4 months was introduced between AVADO and RIBBON-2 by moving the marker for the final AVADO datapoint 2 months later, and 2 months earlier for RIBBON-2.

**Figure S4**. Modified timeline plot showing precision, measured as the inverse of SE/ln(HR)


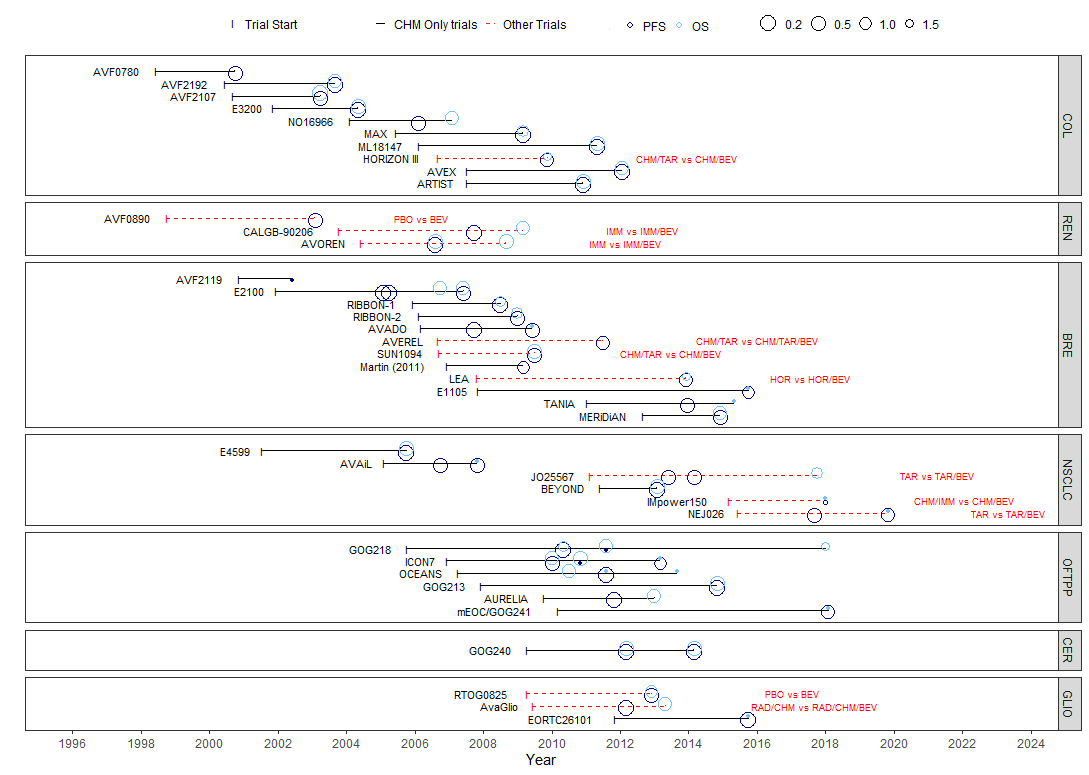


Precisions under 1/2 = 0.5 are plotted as solid black points instead of sized circles. An arbitrary gap of 3-4 months was added to improve visibility, where necessary. 1) a gap 4 months was introduced between AVADO and RIBBON-2 by moving the marker for the final AVADO datapoint 2 months later, and 2 months earlier for RIBBON-2. 2) The first datapoints for GOG218, ICON7 and OCEAN were separated by moving the marker for GOG218 3 months earlier, the marker for ICON7 1 month earlier and the marker for OCEANS 2 months earlier. The final OCEANS datapoint was also plotted 2 month later to not overlap with the final ICON7 marker. 3) The second datapoint for AvaGlio was plotted 3 months later to space it further from marker for RTOG0825.

**Figure S5**. Ridgeline plots of studies ranked by largest OS
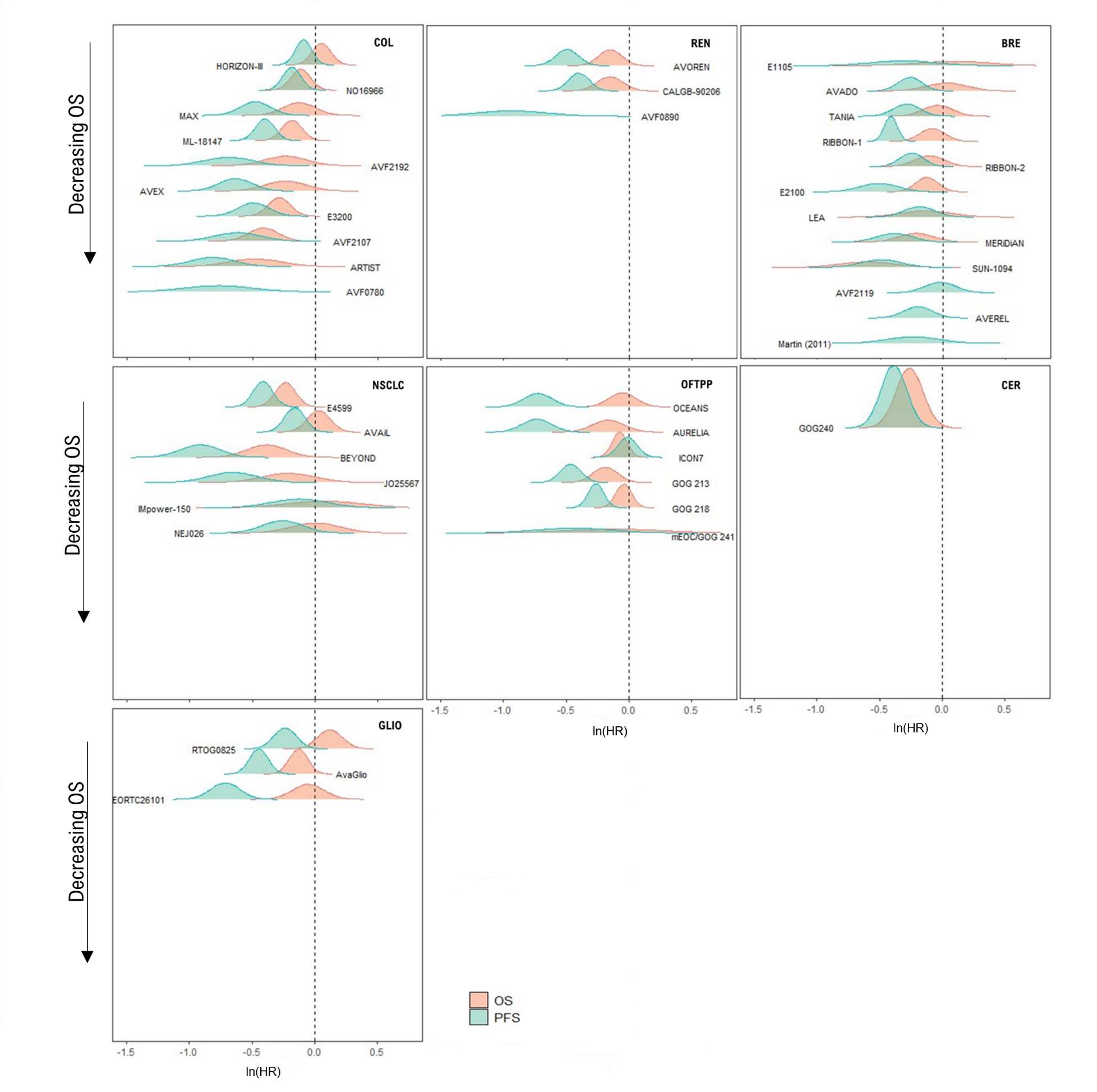


**Figure S6.** Split-violin plots with results for breast cancer synthesis conducted at three pre-specified timepoints using the independent parameter (IP) and common parameter (CP) models for OS and PFS


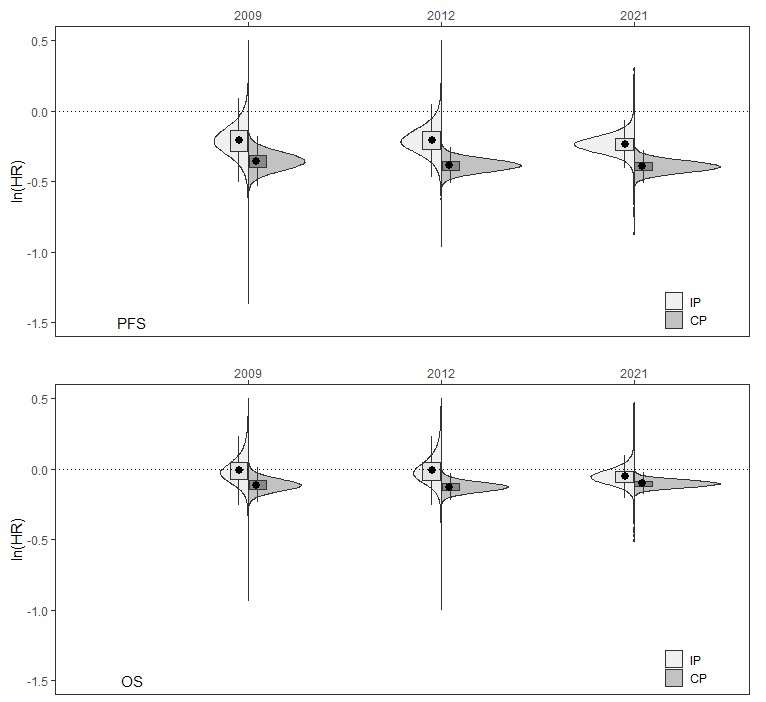


**Abbreviations: CP, common parameter; HR, hazard ratio; IP, independent parameter; OS, overall survival; PFS, progression-free survival.**

# E: Additional figures for Ovarian Cancer as the target indication

When ovarian cancer is the target indication, assuming all studies for all indications available at each selected time point (2011, 2015, 2021) are appropriate for inclusion, ridgeline plots in Figure S7 display results from the IP and CP model at the three analysis time points, for OS and PFS. Split-violin plots displaying the same results are reported in Figure S8. Tables with all results, including estimated treatment effects, heterogeneity and model fit statistics are presented in Section B-IV.

At timepoint 1 (2011) evidence for ovarian cancer consisted only of three studies which inform the IP model for OS, but evidence from 17 trials from 4 other indications are also included in the CP model. The IP model for PFS was informed by 4 studies on ovarian cancer, whereas the CP model was informed by 22 studies over 4 indications. Thus, estimates from the CP model are more precise than for the IP model for both outcomes. The estimates for PFS are much less precise than OS at all timepoints in the indication.

At the second time point (2015), four ovarian cancer studies are included in the synthesis for OS, and a further 27 studies from 6 indications inform the CP model which gives slightly more precise results than at timepoint 1. At the latest timepoint, OS results for both models are more precise. Overall, results from the IP model are the least precise, which is expected as only indication-specific evidence is included in the synthesis.

**Figure S7.** Ridgeline plots with results for ovarian cancer synthesis conducted at three pre-specified timepoints using the IP and CP models for OS and PFS.


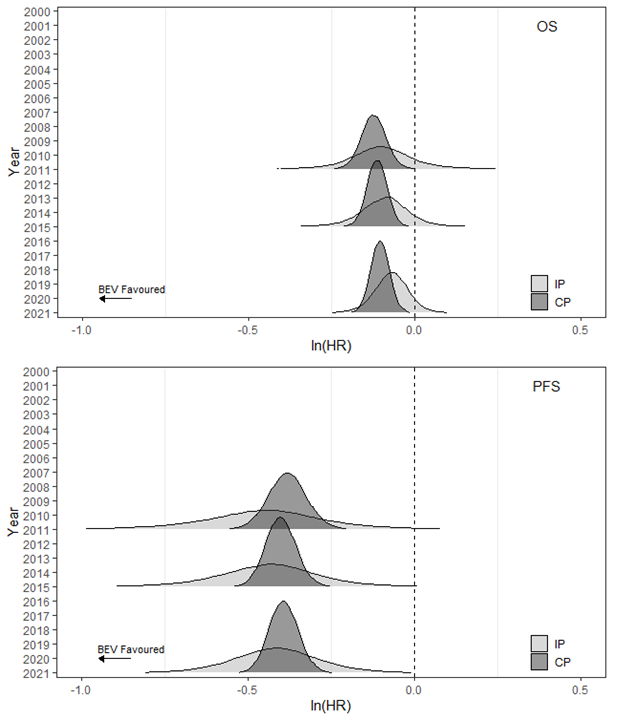


**Abbreviations:** BEV, bevacizumab; CP, common parameter, HR, hazard ratio; IP, independent parameter; PFS, progression-free survival; OS, overall survival

**Figure S8.** Split-violin plots with results for ovarian cancer synthesis conducted at three pre-specified timepoints using the independent parameter (IP) and common parameter (CP) models for OS and PFS


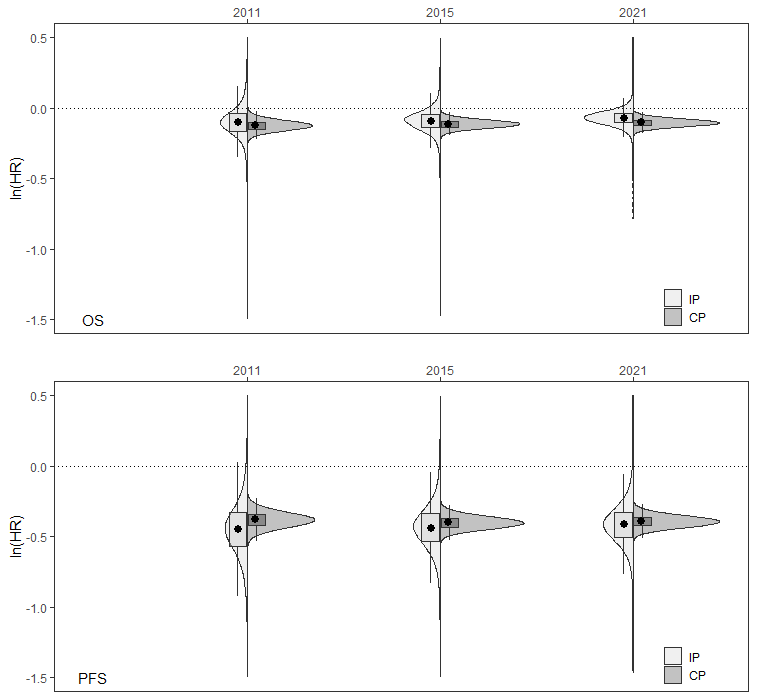


**Abbreviations:** CP, common parameter; HR, hazard ratio; IP, independent parameter; OS, overall survival; PFS, progression-free survival.

# References

1. Miller KD, Chap LI, Holmes FA, et al. Randomized phase III trial of capecitabine compared with bevacizumab plus capecitabine in patients with previously treated metastatic breast cancer. J Clin Oncol 2005; 23: 792-799. DOI: [10.1200/jco.2005.05.098](https://doi.org/10.1200/jco.2005.05.098).

2. Miller KD, Wang M, Gralow J, et al. Paclitaxel plus bevacizumab versus paclitaxel alone for metastatic breast cancer. New England Journal of Medicine 2007; 357: 2666-2676. DOI: [10.1056/NEJMoa072113](https://doi.org/10.1056/NEJMoa072113).

3. Cameron D. Bevacizumab in the first-line treatment of metastatic breast cancer. European Journal of Cancer Supplements 2008; 6: 21-28. DOI: [10.1016/S1359-6349(08)70289-1](https://doi.org/10.1016/S1359-6349(08)70289-1).

4. Robert NJ, Diéras V, Glaspy J, et al. RIBBON-1: Randomized, double-blind, placebo-controlled, phase III trial of chemotherapy with or without bevacizumab for first-line treatment of human epidermal growth factor receptor 2-negative, locally recurrent or metastatic breast cancer. J Clin Oncol 2011; 29: 1252-1260. 20110307. DOI: [10.1200/jco.2010.28.0982](https://doi.org/10.1200/jco.2010.28.0982).

5. Brufsky AM, Hurvitz S, Perez EA, et al. RIBBON-2: A randomized, double-blind, placebo-controlled, phase III trial evaluating the efficacy and safety of bevacizumab in combination with chemotherapy for second-line treatment of human epidermal growth factor receptor 2-negative metastatic breast cancer. J Clin Oncol 2011; 29: 4286-4293. 20111011. DOI: [10.1200/jco.2010.34.1255](https://doi.org/10.1200/jco.2010.34.1255).

6. Miles DW, Chan A, Dirix LY, et al. Phase III study of bevacizumab plus docetaxel compared with placebo plus docetaxel for the first-line treatment of human epidermal growth factor receptor 2–negative metastatic breast cancer. Journal of Clinical Oncology 2010; 28: 3239-3247. DOI: [10.1200/jco.2008.21.6457](https://doi.org/10.1200/jco.2008.21.6457).

7. Miles DW, de Haas SL, Dirix LY, et al. Biomarker results from the AVADO phase 3 trial of first-line bevacizumab plus docetaxel for HER2-negative metastatic breast cancer. Br J Cancer 2013; 108: 1052-1060. 20130219. DOI: [10.1038/bjc.2013.69](https://doi.org/10.1038/bjc.2013.69).

8. Gianni L, Romieu GH, Lichinitser M, et al. AVEREL: A randomized phase III trial evaluating bevacizumab in combination with docetaxel and trastuzumab as first-line therapy for HER2-positive locally recurrent/metastatic breast cancer. J Clin Oncol 2013; 31: 1719-1725. 20130408. DOI: [10.1200/jco.2012.44.7912](https://doi.org/10.1200/jco.2012.44.7912).

9. Robert NJ, Saleh MN, Paul D, et al. Sunitinib plus paclitaxel versus bevacizumab plus paclitaxel for first-line treatment of patients with advanced breast cancer: A phase III, randomized, open-label trial. Clin Breast Cancer 2011; 11: 82-92. 20110411. DOI: [10.1016/j.clbc.2011.03.005](https://doi.org/10.1016/j.clbc.2011.03.005).

10. Martín M, Roche H, Pinter T, et al. Motesanib, or open-label bevacizumab, in combination with paclitaxel, as first-line treatment for HER2-negative locally recurrent or metastatic breast cancer: A phase 2, randomised, double-blind, placebo-controlled study. Lancet Oncol 2011; 12: 369-376. 20110321. DOI: [10.1016/s1470-2045(11)70037-7](https://doi.org/10.1016/s1470-2045(11)70037-7).

11. Martín M, Loibl S, von Minckwitz G, et al. Phase III trial evaluating the addition of bevacizumab to endocrine therapy as first-line treatment for advanced breast cancer: the letrozole/fulvestrant and avastin (LEA) study. J Clin Oncol 2015; 33: 1045-1052. 20150217. DOI: [10.1200/jco.2014.57.2388](https://doi.org/10.1200/jco.2014.57.2388).

12. Arteaga CL, Mayer IA, O'Neill AM, et al. A randomized phase III double-blinded placebo-controlled trial of first-line chemotherapy and trastuzumab with or without bevacizumab for patients with HER2/neu-overexpressing metastatic breast cancer (HER2+ MBC): A trial of the Eastern Cooperative Oncology Group (E1105). Journal of Clinical Oncology 2012; 30: 605-605. DOI: [10.1200/jco.2012.30.15_suppl.605](https://doi.org/10.1200/jco.2012.30.15_suppl.605).

13. A Randomized Phase III Double-Blind Placebo-Controlled Trial of First-Line Chemotherapy and Trastuzumab With or Without Bevacizumab for Patients With HER-2/NEU Over-Expressing Metastatic Breast Cancer. 2007.

14. von Minckwitz G, Puglisi F, Cortes J, et al. Bevacizumab plus chemotherapy versus chemotherapy alone as second-line treatment for patients with HER2-negative locally recurrent or metastatic breast cancer after first-line treatment with bevacizumab plus chemotherapy (TANIA): an open-label, randomised phase 3 trial. Lancet Oncol 2014; 15: 1269-1278. 20140928. DOI: [10.1016/s1470-2045(14)70439-5](https://doi.org/10.1016/s1470-2045(14)70439-5)

15. Vrdoljak E, Marschner N, Zielinski C, et al. Final results of the TANIA randomised phase III trial of bevacizumab after progression on first-line bevacizumab therapy for HER2-negative locally recurrent/metastatic breast cancer. Ann Oncol 2016; 27: 2046-2052. 20160808. DOI: [10.1093/annonc/mdw316](https://doi.org/10.1093/annonc/mdw316).

16. Miles DW, Cameron D, Bondarenko I, et al. Bevacizumab plus paclitaxel versus placebo plus paclitaxel as first-line therapy for HER2-negative metastatic breast cancer (MERiDiAN): A double-blind placebo-controlled randomised phase III trial with prospective biomarker evaluation. Eur J Cancer 2017; 70: 146-155. 20161104. DOI: [10.1016/j.ejca.2016.09.024](https://doi.org/10.1016/j.ejca.2016.09.024).

17. Tewari KS, Sill MW, Long HJr, et al. Improved survival with bevacizumab in advanced cervical cancer. N Engl J Med 2014; 370: 734-743. DOI: [10.1056/NEJMoa1309748](https://doi.org/10.1056/NEJMoa1309748).

18. Tewari KS, Sill MW, Penson RT, et al. Bevacizumab for advanced cervical cancer: final overall survival and adverse event analysis of a randomised, controlled, open-label, phase 3 trial (Gynecologic Oncology Group 240). Lancet 2017; 390: 1654-1663. 20170727. DOI: [10.1016/s0140-6736(17)31607-0](https://doi.org/10.1016/s0140-6736(17)31607-0).

19. Kabbinavar FF, Hurwitz HI, Fehrenbacher L, et al. Phase II, randomized trial comparing bevacizumab plus fluorouracil (FU)/leucovorin (LV) with FU/LV alone in patients with metastatic colorectal cancer. J Clin Oncol 2003; 21: 60-65. DOI: [10.1200/jco.2003.10.066](https://doi.org/10.1200/jco.2003.10.066).

20. Kabbinavar FF, Schulz J, McCleod M, et al. Addition of bevacizumab to bolus fluorouracil and leucovorin in first-line metastatic colorectal cancer: results of a randomized phase II trial. J Clin Oncol 2005; 23: 3697-3705. 20050228. DOI: [10.1200/jco.2005.05.112](https://doi.org/10.1200/jco.2005.05.112).

21. Hurwitz HI, Fehrenbacher L, Novotny WF, et al. Bevacizumab plus irinotecan, fluorouracil, and leucovorin for metastatic colorectal cancer. N Engl J Med 2004; 350: 2335-2342. DOI: [10.1056/NEJMoa032691](https://doi.org/10.1056/NEJMoa032691).

22. Giantonio BJ, Catalano PJ, Meropol NJ, et al. Bevacizumab in combination with oxaliplatin, fluorouracil, and leucovorin (FOLFOX4) for previously treated metastatic colorectal cancer: results from the Eastern Cooperative Oncology Group Study E3200. J Clin Oncol 2007; 25: 1539-1544. DOI: [10.1200/jco.2006.09.6305](https://doi.org/10.1200/jco.2006.09.6305).

23. Saltz LB, Clarke S, Díaz-Rubio E, et al. Bevacizumab in combination with oxaliplatin-based chemotherapy as first-line therapy in metastatic colorectal cancer: a randomized phase III study. J Clin Oncol 2008; 26: 2013-2019. DOI: [10.1200/jco.2007.14.9930](https://doi.org/10.1200/jco.2007.14.9930).

24. Cassidy J, Clarke S, Díaz-Rubio E, et al. XELOX vs FOLFOX-4 as first-line therapy for metastatic colorectal cancer: NO16966 updated results. Br J Cancer 2011; 105: 58-64. 20110614. DOI: [10.1038/bjc.2011.201](https://doi.org/10.1038/bjc.2011.201).

25. Tebbutt NC, Wilson K, Gebski VJ, et al. Capecitabine, Bevacizumab, and Mitomycin in First-Line Treatment of Metastatic Colorectal Cancer: Results of the Australasian Gastrointestinal Trials Group Randomized Phase III MAX Study. Journal of Clinical Oncology 2010; 28: 3191-3198. DOI: [10.1200/jco.2009.27.7723](https://doi.org/10.1200/jco.2009.27.7723).

26. Bennouna J, Sastre J, Arnold D, et al. Continuation of bevacizumab after first progression in metastatic colorectal cancer (ML18147): a randomised phase 3 trial. Lancet Oncol 2013; 14: 29-37. 20121116. DOI: [10.1016/s1470-2045(12)70477-1](https://doi.org/10.1016/s1470-2045(12)70477-1).

27. Kubicka S, Greil R, André T, et al. Bevacizumab plus chemotherapy continued beyond first progression in patients with metastatic colorectal cancer previously treated with bevacizumab plus chemotherapy: ML18147 study KRAS subgroup findings. Ann Oncol 2013; 24: 2342-2349. 20130712. DOI: [10.1093/annonc/mdt231](https://doi.org/10.1093/annonc/mdt231).

28. Schmoll H-J, Cunningham D, Sobrero A, et al. Cediranib with mFOLFOX6 versus bevacizumab with mFOLFOX6 as first-line treatment for patients with advanced colorectal cancer: A double-blind, randomized phase III study (HORIZON III). J Clin Oncol 2012; 30: 3588-3595. 20120910. DOI: [10.1200/jco.2012.42.5355](https://doi.org/10.1200/jco.2012.42.5355).

29. Cunningham D, Lang I, Marcuello E, et al. Bevacizumab plus capecitabine versus capecitabine alone in elderly patients with previously untreated metastatic colorectal cancer (AVEX): an open-label, randomised phase 3 trial. Lancet Oncol 2013; 14: 1077-1085. 20130910. DOI: [10.1016/s1470-2045(13)70154-2](https://doi.org/10.1016/s1470-2045(13)70154-2).

30. Guan Z-Z, Xu J-M, Luo R-C, et al. Efficacy and safety of bevacizumab plus chemotherapy in Chinese patients with metastatic colorectal cancer: a randomized phase III ARTIST trial. Chin J Cancer 2011; 30: 682-689. DOI: [10.5732/cjc.011.10188](https://doi.org/10.5732/cjc.011.10188).

31. Gilbert MR, Dignam JJ, Armstrong TS, et al. A randomized trial of bevacizumab for newly diagnosed glioblastoma. N Engl J Med 2014; 370: 699-708. DOI: [10.1056/NEJMoa1308573](https://doi.org/10.1056/NEJMoa1308573).

32. Sandmann T, Bourgon R, Garcia J, et al. Patients with proneural glioblastoma may derive overall survival benefit from the addition of bevacizumab to first-line radiotherapy and temozolomide: Retrospective analysis of the AVAglio trial. J Clin Oncol 2015; 33: 2735-2744. 20150629. DOI: [10.1200/jco.2015.61.5005](https://doi.org/10.1200/jco.2015.61.5005).

33. Wick W, Brandes AA, Gorlia T, et al. EORTC 26101 phase III trial exploring the combination of bevacizumab and lomustine in patients with first progression of a glioblastoma. Journal of Clinical Oncology 2016; 34: 2001-2001. DOI: [10.1200/JCO.2016.34.15_suppl.2001](https://doi.org/10.1200/JCO.2016.34.15_suppl.2001).

34. Sandler A, Gray R, Perry MC, et al. Paclitaxel-carboplatin alone or with bevacizumab for non-small-cell lung cancer. N Engl J Med 2006; 355: 2542-2550. DOI: [10.1056/NEJMoa061884](https://doi.org/10.1056/NEJMoa061884).

35. Reck M, von Pawel J, Zatloukal P, et al. Phase III trial of cisplatin plus gemcitabine with either placebo or bevacizumab as first-line therapy for nonsquamous non-small-cell lung cancer: AVAil. J Clin Oncol 2009; 27: 1227-1234. 20090202. DOI: [10.1200/jco.2007.14.5466](https://doi.org/10.1200/jco.2007.14.5466).

36. Reck M, von Pawel J, Zatloukal P, et al. Overall survival with cisplatin-gemcitabine and bevacizumab or placebo as first-line therapy for nonsquamous non-small-cell lung cancer: Results from a randomised phase III trial (AVAiL). Ann Oncol 2010; 21: 1804-1809. 20100211. DOI: [10.1093/annonc/mdq020](https://doi.org/10.1093/annonc/mdq020).

37. Seto T, Kato T, Nishio M, et al. Erlotinib alone or with bevacizumab as first-line therapy in patients with advanced non-squamous non-small-cell lung cancer harbouring EGFR mutations (JO25567): An open-label, randomised, multicentre, phase 2 study. Lancet Oncol 2014; 15: 1236-1244. 20140827. DOI: [10.1016/s1470-2045(14)70381-x](https://doi.org/10.1016/s1470-2045(14)70381-x).

38. Yamamoto N, Seto T, Nishio M, et al. Erlotinib plus bevacizumab vs erlotinib monotherapy as first-line treatment for advanced EGFR mutation-positive non-squamous non-small-cell lung cancer: Survival follow-up results of the randomized JO25567 study. Lung Cancer 2021; 151: 20-24. 20201120. DOI: [10.1016/j.lungcan.2020.11.020](https://doi.org/10.1016/j.lungcan.2020.11.020).

39. Zhou C, Wu Y-L, Chen G, et al. BEYOND: A Randomized, Double-Blind, Placebo-Controlled, Multicenter, Phase III Study of First-Line Carboplatin/Paclitaxel Plus Bevacizumab or Placebo in Chinese Patients With Advanced or Recurrent Nonsquamous Non-Small-Cell Lung Cancer. J Clin Oncol 2015; 33: 2197-2204. 20150526. DOI: [10.1200/jco.2014.59.4424](https://doi.org/10.1200/jco.2014.59.4424).

40. Reck M, Mok TSK, Nishio M, et al. Atezolizumab plus bevacizumab and chemotherapy in non-small-cell lung cancer (IMpower150): Key subgroup analyses of patients with EGFR mutations or baseline liver metastases in a randomised, open-label phase 3 trial. Lancet Respir Med 2019; 7: 387-401. 20190325. DOI: [10.1016/s2213-2600(19)30084-0](https://doi.org/10.1016/s2213-2600(19)30084-0).

41. Socinski MA, Nishio M, Jotte RM, et al. IMpower150 Final Overall Survival Analyses for Atezolizumab Plus Bevacizumab and Chemotherapy in First-Line Metastatic Nonsquamous NSCLC. J Thorac Oncol 2021; 16: 1909-1924. 20210724. DOI: [10.1016/j.jtho.2021.07.009](https://doi.org/10.1016/s2213-2600(19)30084-0).

42. Saito H, Fukuhara T, Furuya N, et al. Erlotinib plus bevacizumab versus erlotinib alone in patients with EGFR-positive advanced non-squamous non-small-cell lung cancer (NEJ026): Interim analysis of an open-label, randomised, multicentre, phase 3 trial. Lancet Oncol 2019; 20: 625-635. 20190408. DOI: [10.1016/s1470-2045(19)30035-x](https://doi.org/10.1016/s1470-2045(19)30035-x).

43. Kawashima Y, Fukuhara T, Saito H, et al. Bevacizumab plus erlotinib versus erlotinib alone in Japanese patients with advanced, metastatic, EGFR-mutant non-small-cell lung cancer (NEJ026): Overall survival analysis of an open-label, randomised, multicentre, phase 3 trial. Lancet Respir Med 2022; 10: 72-82. 20210826. DOI: [10.1016/s2213-2600(21)00166-1](https://doi.org/10.1016/s2213-2600(21)00166-1).

44. Burger RA, Brady MF, Bookman MA, et al. Incorporation of bevacizumab in the primary treatment of ovarian cancer. N Engl J Med 2011; 365: 2473-2483. DOI: [10.1056/NEJMoa1104390](https://doi.org/10.1056/NEJMoa1104390).

45. Tewari KS, Burger RA, Enserro D, et al. Final Overall Survival of a Randomized Trial of Bevacizumab for Primary Treatment of Ovarian Cancer. J Clin Oncol 2019; 37: 2317-2328. 20190619. DOI: [10.1200/jco.19.01009](https://doi.org/10.1200/jco.19.01009).

46. Perren TJ, Swart AM, Pfisterer J, et al. A phase 3 trial of bevacizumab in ovarian cancer. N Engl J Med 2011; 365: 2484-2496. DOI: [10.1056/NEJMoa1103799](https://doi.org/10.1056/NEJMoa1103799).

47. Oza AM, Cook AD, Pfisterer J, et al. Standard chemotherapy with or without bevacizumab for women with newly diagnosed ovarian cancer (ICON7): Overall survival results of a phase 3 randomised trial. Lancet Oncol 2015; 16: 928-936. 20150623. DOI: [10.1016/s1470-2045(15)00086-8](https://doi.org/10.1016/s1470-2045(15)00086-8).

48. Aghajanian C, Blank SV, Goff BA, et al. OCEANS: A randomized, double-blind, placebo-controlled phase III trial of chemotherapy with or without bevacizumab in patients with platinum-sensitive recurrent epithelial ovarian, primary peritoneal, or fallopian tube cancer. J Clin Oncol 2012; 30: 2039-2045. 20120423. DOI: [10.1200/jco.2012.42.0505](https://doi.org/10.1200/jco.2012.42.0505).

49. Aghajanian C, Goff BA, Nycum LR, et al. Final overall survival and safety analysis of OCEANS, a phase 3 trial of chemotherapy with or without bevacizumab in patients with platinum-sensitive recurrent ovarian cancer. Gynecol Oncol 2015; 139: 10-16. 20150810. DOI: [10.1016/j.ygyno.2015.08.004](https://doi.org/110.1016/j.ygyno.2015.08.004).

50. Coleman RL, Brady MF, Herzog TJ, et al. Bevacizumab and paclitaxel-carboplatin chemotherapy and secondary cytoreduction in recurrent, platinum-sensitive ovarian cancer (NRG Oncology/Gynecologic Oncology Group study GOG-0213): a multicentre, open-label, randomised, phase 3 trial. Lancet Oncol 2017; 18: 779-791. 20170421. DOI: [10.1016/s1470-2045(17)30279-6](https://doi.org/10.1016/s1470-2045(17)30279-6).

51. Pujade-Lauraine E, Hilpert F, Weber B, et al. Bevacizumab combined with chemotherapy for platinum-resistant recurrent ovarian cancer: The AURELIA open-label randomized phase III trial. J Clin Oncol 2014; 32: 1302-1308. 20140317. DOI: [10.1200/jco.2013.51.4489](https://doi.org/10.1200/jco.2013.51.4489).

52. Bamias A, Gibbs E, Khoon Lee C, et al. Bevacizumab with or after chemotherapy for platinum-resistant recurrent ovarian cancer: exploratory analyses of the AURELIA trial. Ann Oncol 2017; 28: 1842-1848. DOI: [10.1093/annonc/mdx228](https://doi.org/10.1093/annonc/mdx228).

53. Gore M, Hackshaw A, Brady WE, et al. An international, phase III randomized trial in patients with mucinous epithelial ovarian cancer (mEOC/GOG 0241) with long-term follow-up: and experience of conducting a clinical trial in a rare gynecological tumor. Gynecol Oncol 2019; 153: 541-548. 20190418. DOI: [10.1016/j.ygyno.2019.03.256](https://doi.org/10.1016/j.ygyno.2019.03.256).

54. Yang JC, Haworth L, Sherry RM, et al. A randomized trial of bevacizumab, an anti-vascular endothelial growth factor antibody, for metastatic renal cancer. N Engl J Med 2003; 349: 427-434. DOI: [10.1056/NEJMoa021491](https://doi.org/10.1056/NEJMoa021491).

55. Rini BI, Halabi S, Rosenberg JE, et al. Bevacizumab plus interferon alfa compared with interferon alfa monotherapy in patients with metastatic renal cell carcinoma: CALGB 90206. J Clin Oncol 2008; 26: 5422-5428. 20081020. DOI: [10.1200/jco.2008.16.9847](https://doi.org/10.1200/jco.2008.16.9847).

56. Rini BI, Halabi S, Rosenberg JE, et al. Phase III trial of bevacizumab plus interferon alfa versus interferon alfa monotherapy in patients with metastatic renal cell carcinoma: final results of CALGB 90206. J Clin Oncol 2010; 28: 2137-2143. 20100405. DOI: [10.1200/jco.2009.26.5561](https://doi.org/10.1200/jco.2009.26.5561).

57. Escudier B, Pluzanska A, Koralewski P, et al. Bevacizumab plus interferon alfa-2a for treatment of metastatic renal cell carcinoma: a randomised, double-blind phase III trial. Lancet 2007; 370: 2103-2111. DOI: [10.1016/s0140-6736(07)61904-7](https://doi.org/10.1016/s0140-6736(07)61904-7).

58. Escudier B, Bellmunt J, Négrier S, et al. Phase III trial of bevacizumab plus interferon alfa-2a in patients with metastatic renal cell carcinoma (AVOREN): final analysis of overall survival. J Clin Oncol 2010; 28: 2144-2150. 20100405. DOI: [10.1200/jco.2009.26.7849](https://doi.org/10.1200/jco.2009.26.7849).

59. Shen L, Li J, Xu J, et al. Bevacizumab plus capecitabine and cisplatin in Chinese patients with inoperable locally advanced or metastatic gastric or gastroesophageal junction cancer: Randomized, double-blind, phase III study (AVATAR study). Gastric Cancer 2015; 18: 168-176. 20140221. DOI: [10.1007/s10120-014-0351-5](https://doi.org/10.1007/s10120-014-0351-5).

60. Ohtsu A, Shah MA, Van Cutsem E, et al. Bevacizumab in combination with chemotherapy as first-line therapy in advanced gastric cancer: A randomized, double-blind, placebo-controlled phase III study. J Clin Oncol 2011; 29: 3968-3976. 20110815. DOI: [10.1200/jco.2011.36.2236](https://doi.org/10.1200/jco.2011.36.2236)

61. Seymour JF, Pfreundschuh M, Trnĕný M, et al. R-CHOP with or without bevacizumab in patients with previously untreated diffuse large B-cell lymphoma: Final MAIN study outcomes. Haematologica 2014; 99: 1343-1349. 20140603. DOI: [10.3324/haematol.2013.100818](https://doi.org/10.3324/haematol.2013.100818).

62. Rosenberg JE, Ballman KA, Halabi S, et al. Randomized Phase III Trial of Gemcitabine and Cisplatin With Bevacizumab or Placebo in Patients With Advanced Urothelial Carcinoma: Results of CALGB 90601 (Alliance). J Clin Oncol 2021; 39: 2486-2496. 20210514. DOI: [10.1200/jco.21.00286](https://doi.org/10.1200/jco.21.00286).

63. Kelly WK, Halabi S, Carducci M, et al. Randomized, Double-Blind, Placebo-Controlled Phase III Trial Comparing Docetaxel and Prednisone With or Without Bevacizumab in Men With Metastatic Castration-Resistant Prostate Cancer: CALGB 90401. Journal of Clinical Oncology 2012; 30: 1534-1540. DOI: [10.1200/jco.2011.39.4767](https://doi.org/10.1200/jco.2011.39.4767).

64. Hensley ML, Miller A, O'Malley DM, et al. Randomized phase III trial of gemcitabine plus docetaxel plus bevacizumab or placebo as first-line treatment for metastatic uterine leiomyosarcoma: an NRG Oncology/Gynecologic Oncology Group study. J Clin Oncol 2015; 33: 1180-1185. 20150223. DOI: [10.1200/jco.2014.58.3781](https://doi.org/10.1200/jco.2014.58.3781).

65. Sutton AJ and Abrams KR. Bayesian methods in meta-analysis and evidence synthesis. Statistical methods in medical research 2001; 10: 277-303.

66. Röver C, Bender R, Dias S, et al. On weakly informative prior distributions for the heterogeneity parameter in Bayesian random‐effects meta‐analysis. Research Synthesis Methods 2021; 12: 448-474.
